# Collaborative penetration testing suite for emerging generative AI algorithms

**DOI:** 10.1007/s10489-025-06908-1

**Published:** 2025-10-16

**Authors:** Petar Radanliev

**Affiliations:** 1 Department of Computer Science, University of Oxford, Wolfson Building, Parks Rd, OX1 3QG Oxford, England; 2 The Alan Turing Institute, British Library, 96 Euston Rd, NW1 2DB London, UK; 3 University of the West of England Bristol, Coldharbour Ln, Stoke Gifford, Bristol, BS16 1QY UK

**Keywords:** Generative artificial intelligence security, Quantum-Resistant cryptography, Dynamic application security testing, Blockchain-Enhanced logging, Advanced persistent threats

## Abstract

Generative artificial intelligence systems remain vulnerable to sophisticated cyber threats and the emerging challenges posed by quantum computing. This study proposes and evaluates a new penetration testing suite to address quantum security concerns. The suite integrates dynamic and static application security testing (DAST and SAST) using OWASP ZAP, Burp Suite, SonarQube, and Fortify to detect and resolve vulnerabilities across application lifecycles. Real-time monitoring through interactive application security testing (IAST) with Contrast Assess near-real-time analysis facilitates pre-emptive remediation and remediation of insecure data handling and encryption flaws. Blockchain-enhanced logging, implemented via Hyperledger Fabric, provides tamper-proof and auditable records of all security activities. Furthermore, quantum-resistant cryptographic protocols, including lattice-based cryptography and RLWE, safeguard against quantum decryption threats, validated through simulated quantum attack scenarios. AI-driven red team simulations emulate adversarial and quantum-assisted attacks, uncovering vulnerabilities overlooked by traditional methods. Key results include the identification and remediation of over 300 vulnerabilities, a 70% reduction in high-severity issues within two weeks of testing, and a 90% resolution efficiency for blockchain-logged vulnerabilities. Quantum-resistant protocols exhibited strong resilience under adversarial conditions against simulated quantum attacks, achieving secure API encryption and data transmission. This research establishes a new protocol for securing generative AI systems, combining advanced tools, methodologies, and industry-tested methods.

## Introduction

Generative artificial intelligence (AI) algorithms and models [1], such as Generative Adversarial Networks (GANs) [2] and Large Language Models (LLMs) [3], possess highly capable of synthesising domain-specific data representations, simulate intricate systems, and perform complex tasks traditionally reserved for human experts. Their growing use in sensitive domains has introduced significant security concerns. These systems are increasingly targeted by sophisticated attacks that exploit inherent vulnerabilities, creating risks to privacy, operational reliability, and the integrity of the systems they underpin. The challenges are further compounded by the rise of quantum computing, which threatens the reliability of existing cryptographic safeguards.

Generative AI systems are susceptible to several attack vectors, such as model inversion, data poisoning, and adversarial inputs. For example, adversarial inputs can cause these models to produce incorrect results or exhibit undesirable behaviours, potentially leading to harmful outcomes. Model inversion techniques enable attackers to extract sensitive training data, posing serious risks to privacy. Data poisoning attacks compromise training datasets, altering the functionality of the model in unintended ways. These threats are particularly concerning in critical applications, including healthcare and financial systems, where such vulnerabilities could lead to severe consequences.

The emergence of quantum computing intensifies these issues by introducing the capability to break widely used encryption algorithms such as RSA and elliptic curve cryptography. Algorithms like Shor’s algorithm can factorise large numbers exponentially faster than classical techniques, rendering many established security measures ineffective. This requires the development of security measures that can withstand not only today’s threats but also the computational challenges posed by quantum systems.

This study addresses these issues by introducing a penetration testing suite designed to comprehensively evaluate and enhance the security of generative AI systems. The suite incorporates dynamic and static application security testing (DAST & SAST) to detect vulnerabilities during runtime and development stages. It also uses blockchain-enhanced logging to create tamper-proof records of security events, ensuring accountability and transparency. To safeguard against quantum threats, the framework integrates quantum-resistant cryptographic protocols, such as lattice-based cryptography and RLWE, which provide robust protection for sensitive data and communications. Additionally, AI-driven red team simulations were employed to emulate advanced threats, including adversarial attacks and quantum-assisted exploits, enabling the identification of vulnerabilities that traditional techniques might overlook.

The paper details the related work, methodologies, and evaluation of this framework. Key findings include a 70% reduction in high-priority vulnerabilities within two weeks of implementation and resilience to simulated quantum attacks, demonstrating the framework’s effectiveness in addressing both existing and future challenges. The study offers an operationally viable security framework for improving the security of AI systems while offering tools and methods that can be adapted to other domains requiring advanced cybersecurity measures.

## Literature review

Generative AI systems are vulnerable to model inversion attacks, data poisoning, and adversarial examples, hence, the literature review starts with specific studies on these specific vulnerabilities. Model inversion attacks exploit the model to reconstruct sensitive training data, thereby breaching data privacy [4]. Data poisoning involves the injection of malicious data into the training set, corrupting the model’s outputs [5]. Adversarial examples, on the other hand, introduce subtle perturbations to input data, deceiving the model into making erroneous predictions [6, 7].

The emergence of quantum computing [8] further worsens these challenges by threatening the foundational cryptographic schemes [9] that underpin current cybersecurity infrastructures [10]. Quantum algorithms, notably Shor’s algorithm [11], can factorise large integers exponentially faster than classical algorithms, thereby compromising widely used encryption schemes such as RSA [12] and elliptic curve cryptography [11]. This looming threat necessitates the development and implementation of quantum-resistant cryptographic protocols. Lattice-based cryptography [13] has emerged as a solution, using mathematical problems that are resistant to quantum attacks [13]. Similarly, Ring Learning with Errors (RLWE) [14] offers robustness against quantum decryption techniques. Integrating these quantum-resistant protocols is critical to ensuring the security of sensitive data in a post-quantum era.

Security testing methodologies, such as the ‘Dynamic Application Security Testing’ (DAST) tools [15, 16], such as OWASP Zed Attack Proxy [17] and Burp Suite [18–20], are instrumental in identifying vulnerabilities in running applications by simulating various attack scenarios [17]. In contrast, Static Application Security Testing (SAST) tools, including SonarQube and Fortify, perform in-depth source code analysis to detect security flaws at the code level [21]. Interactive Application Security Testing (IAST) synthesises the strengths of both DAST and SAST by providing real-time insights into security vulnerabilities during the development process, ensuring that security is integrated from the ground up. These methodologies are essential for the robust assessment and fortification of generative AI systems against cyber threats.

In addition to these testing methodologies, blockchain technology offers a solution for ensuring the integrity and immutability of security logs. Hyperledger Fabric, a permissioned blockchain platform, enables the creation of secure, tamper-proof logs for penetration testing activities [22]. Each log entry, encompassing detected vulnerabilities and remediation actions, is immutably recorded on the blockchain, ensuring that records cannot be altered without detection. This level of transparency and accountability is critical for maintaining the veracity of security processes and facilitating compliance with regulatory requirements.

Red team simulations, driven by AI, are another critical component in the cybersecurity resilience assessment. These simulations use machine learning models to emulate advanced persistent threats and other sophisticated attack strategies, objectively evaluating the system’s security posture [2]. AI-driven red team exercises can generate various attack scenarios, including AI-generated phishing attacks and adversarial machine learning attacks, thereby thoroughly evaluating system vulnerabilities. The insights gleaned from these simulations are invaluable in informing the development of effective mitigation strategies and enhancing the overall security framework.

Integrating these advanced methodologies into new security frameworks is also required for addressing the quantum computing challenges of securing autonomous systems.

### Review of the research methodologies applied

To develop a collaborative penetration testing suite for generative AI systems, the study employed a diverse set of security methodologies that collectively provide a defence mechanism. The chosen methodologies: Dynamic and Static Application Security Testing (DAST & SAST), Interactive Application Security Testing (IAST), Blockchain-Enhanced Security Logging, Quantum-Resistant Cryptography, and AI-driven Red Team Simulations, are carefully selected for their ability to address various facets of cybersecurity. These methodologies ensure real-time vulnerability detection, continuous code quality assessment, immutable logging of security events, protection against future quantum decryption threats, and resilience against sophisticated attack strategies. Each method analysed in Table 1 brings unique strengths: DAST and SAST offer immediate and continuous security insights, IAST integrates into the development pipeline to provide ongoing protection, blockchain logging ensures transparency and tamper-proof records, quantum-resistant cryptography secures against emerging quantum threats, and AI-driven simulations test the system’s robustness against advanced persistent threats. Table 1 provides a clear and structured overview of the codification of each methodology, tool, and approach.

**Table 1:** Codification key, organised for clarity and alignment with the research methodologies

Methodology	Tools/Protocols	Approach	Description
1: Dynamic and Static Application Security Testing (DAST & SAST)	OWASP ZAP, Burp Suite, SonarQube, Fortify	**1.1 Dynamic Testing**	Uses OWASP ZAP and Burp Suite for real-time security assessments, including vulnerability scanning, fuzzing, and manual exploitation of applications.
		**1.2 Static Testing**	Employs SonarQube for continuous code quality checks and Fortify for deep static analysis, targeting insecure coding practices and logic errors.
2: Interactive Application Security Testing (IAST)	Contrast Assess	**2.1 CI/CD Integration**	Embeds IAST into the CI/CD pipeline for real-time vulnerability detection during development and runtime.
		**2.2 Focus Areas**	Detects hard-coded secrets, weak encryption algorithms, and vulnerabilities related to unvalidated input fields.
3: Blockchain-Enhanced Security Logging	Hyperledger Fabric	**3.1 Immutable Logging System**	Records penetration testing activities on a tamper-proof blockchain ledger, ensuring transparency and regulatory compliance.
		**3.2 Lifecycle Coverage**	Logs all vulnerabilities, remediation actions, and system changes throughout the penetration testing lifecycle.
4: Quantum-Resistant Cryptography	Lattice-Based Cryptography, Ring Learning with Errors (RLWE)	**4.1 Data Encryption**	Encrypts sensitive information using lattice-based cryptography to safeguard against quantum decryption techniques.
		**4.2 Secure Communications**	Implements RLWE to protect data transmission channels against eavesdropping and interception.
		**4.3 Key Management**	Enhances cryptographic key generation, distribution, and storage using quantum-resistant methods.
5: AI-Driven Red Team Simulations	AI Models for Phishing, Adversarial Attacks, and Quantum Decryption	**5.1 Simulation Design**	Creates evolving attack scenarios using AI-driven models to emulate advanced persistent threats and sophisticated attack techniques.
		**5.2 Evaluation**	Measures success rates, system response times, and resilience to sophisticated and quantum-enhanced threats.

The methodologies reviewed below, are systematically codified and presented in Table 1 to provide a clear and concise overview of their structure, tools, and approaches. Table 1 organises each methodology into its respective tools and detailed approaches, such as Approach 1.1 for real-time dynamic application security testing (DAST) using OWASP ZAP and Burp Suite, and Approach 1.2 for static application security testing (SAST) with SonarQube and Fortify. Similarly, it details the integration of Contrast Assess in the CI/CD pipeline for Interactive Application Security Testing (IAST), the deployment of Hyperledger Fabric for immutable blockchain-enhanced security logging, and the implementation of lattice-based cryptography and RLWE protocols for quantum-resistant encryption. AI-driven red team simulations, emulating advanced persistent threats and quantum-enhanced attack techniques, are also codified to reflect their unique contribution to system security assessments. This structured classification provides consistency and clarity on the methodologies’ scope, tools, and specific applications within the penetration testing suite.

### Review of methodology 1: dynamic and static application security testing (DAST & SAST)

**Tools**: OWASP ZAP [17], Burp Suite, SonarQube, Fortify. Approach 1.2: Dynamic Testing (DAST): Use OWASP ZAP and Burp Suite for real-time security assessments on running applications. These tools simulate various attack scenarios to identify runtime vulnerabilities such as injection flaws, authentication issues, and configuration errors. OWASP ZAP was configured to perform automated scans and identify security issues in web applications, while Burp Suite was used for more detailed manual testing, including fuzzing and brute-force attacks [23]. Real-time testing ensures vulnerabilities are detected during operation, providing immediate feedback for remediation. Specific attack vectors include SQL injection, cross-site scripting (XSS), and cross-site request forgery (CSRF). Approach 1.2: Static Testing (SAST): Employ SonarQube and Fortify to conduct source code analysis. These tools can scan the entire codebase to uncover security flaws like insecure coding practices, logic errors, and potential backdoors [24, 25]. SonarQube was integrated into the CI/CD pipeline to provide continuous analysis and feedback on code quality, while Fortify is used for deep-dive static analysis and remediation. The static analysis is performed at different stages of the development lifecycle to ensure early detection and resolution of vulnerabilities. This dual approach ensures the applications’ immediate and long-term security, targeting vulnerabilities such as buffer overflows, improper error handling, and insufficient logging.

### Review of methodology 2: interactive application security testing (IAST)

 Tool: Contrast Assess. Approach 2.1: Integration with CI/CD Pipeline: Integrate Contrast Assess into the continuous integration/continuous deployment (CI/CD) pipeline to combine the strengths of static and dynamic testing. This tool provides real-time insights into security vulnerabilities as code is written and tested. The tool was used to monitor applications during runtime and to identify insecure data handling, access control weaknesses, and encryption flaws. Emphasis was placed on vulnerabilities related to quantum decryption techniques, ensuring the system is prepared for future threats [26]. By integrating IAST into the development process, security is built into the application from the ground up. Specific focus areas include identifying hard-coded secrets, weak encryption algorithms, and unvalidated input fields.

### Review of methodology 3: blockchain-enhanced security logging

 Implementation: Hyperledger Fabric. Approach 3.1: Immutable Logging System: Deploy Hyperledger Fabric [22] to create an immutable and secure logging system for all penetration testing activities [27]. Blockchain technology was used to ensure that all logs are tamper-proof, providing a transparent audit trail. Each log entry, including detected vulnerabilities, remediation actions, and system changes, was recorded on the blockchain, making it impossible to alter the records without detection. This enhances trust and accountability, ensuring that security processes are verifiable and reliable [28]. The blockchain-enhanced logging system also facilitated compliance with regulatory requirements by providing an immutable record of all security-related activities. This approach covers the entire lifecycle of penetration testing activities, from initial scans to final remediation.

### Review of methodology 4: quantum-resistant cryptography

 Protocols: Lattice-based cryptography, Ring Learning with Errors (RLWE). Approach 4.1: Develop and Implement Protocols: Develop and implement quantum-resistant cryptographic protocols to protect sensitive data against quantum attacks [29]. Lattice-based cryptography [13, 14], 30]– [40] and RLWE [14] was integrated into the security framework to provide robust encryption methods resistant to quantum computing threats. These protocols are applied to critical areas such as data encryption, secure communication channels, and key management processes. The development process involved rigorous mathematical validation and extensive testing to ensure the protocols were secure and efficient [41]. Specific applications include securing RESTful APIs, encrypting database fields, and protecting user authentication mechanisms. By adopting quantum-resistant cryptographic methods, the system was safeguarded against future quantum decryption capabilities, ensuring long-term security of sensitive information [11, 12, 42].

### Review of methodology 5: red team AI simulations

 Exercises: AI-driven attack simulations. Approach 5.1: Conduct Specialised Simulations: Conduct specialised red team simulations using AI-driven models to test the resilience of the system against sophisticated cyber-attack strategies [2]. These simulations emulated advanced persistent threats (APTs) and other high-level attacks, using machine learning models to create realistic and evolving attack scenarios [43]. AI algorithms was used to generate adversarial examples and simulate insider threats. The red team exercises included conventional and quantum-enhanced attack techniques, providing a assessment of the system’s security posture. Specific simulations involved testing against AI-generated phishing attacks, adversarial machine learning attacks, and simulated quantum decryption attempts. The outcomes of these simulations inform the development of mitigation strategies and enhance the overall security framework [44, 45].

### The contribution of each methodology

The Table 2 summarises the methodologies implemented in the penetration testing suite, detailing the specific tools and technologies used alongside their unique contributions to enhancing generative AI system security. Each methodology in Table 2 targets a distinct aspect of the security framework, from runtime and code-level vulnerability detection to advanced cryptographic protocols and AI-driven threat simulations. By integrating these methodologies (see Table 2), the suite addresses conventional cyber threats and emerging risks, such as quantum computing and adversarial attacks, while ensuring transparency, accountability, and long-term resilience against anticipated threats.

**Table 2:** Summary of the contribution of each methodology

Methodology	Tools/Technologies Used	Key Contributions
Dynamic and Static Application Security Testing (DAST & SAST)	OWASP ZAP, Burp Suite, SonarQube, Fortify	- Identifies runtime vulnerabilities (e.g., SQL injection, XSS, CSRF) using DAST. - Detects insecure coding practices, logic errors, and backdoors with SAST. - Provides comprehensive coverage across runtime and code-level vulnerabilities. - Continuous assessment via CI/CD pipeline integration ensures early and proactive remediation.
Interactive Application Security Testing (IAST)	Contrast Assess	- Real-time feedback during application runtime. - Detects vulnerabilities such as hardcoded secrets, weak encryption, and insecure data handling. - Seamless integration into CI/CD pipeline reduces post-deployment risks. - Bridges static and dynamic testing gaps for proactive security.
Blockchain-Enhanced Security Logging	Hyperledger Fabric	- Provides tamper-proof, immutable logs for all security activities. - Facilitates regulatory compliance and secure audit trails. - Enhances transparency, accountability, and trust in penetration testing processes.
Quantum-Resistant Cryptographic Protocols	Lattice-based cryptography, RLWE	- Ensures robust encryption of sensitive data, communication channels, and APIs. - Validated through simulated quantum decryption scenarios. - Future-proofs generative AI systems against quantum threats. - Protects cryptographic keys and long-term data security.
AI-Driven Red Team Simulations	Machine learning models for adversarial attack generation	- Simulates real-world and advanced cyber threats, including AI-generated phishing, adversarial ML attacks, and quantum-assisted breaches. - Identifies vulnerabilities overlooked by traditional methods. - Provides comprehensive evaluations of generative AI systems’ security postures. - Enables iterative improvements to system resilience and defences.

Table 2 highlights the comprehensive nature of the penetration testing suite by demonstrating how each methodology contributes to the overall security framework. The combination of DAST, SAST, and IAST ensures layered coverage across runtime, code, and active application environments, fostering proactive remediation. Blockchain-enhanced logging stands out as a critical innovation, providing immutable records that ensure compliance and build trust in collaborative settings. The integration of quantum-resistant cryptographic protocols future-proofs generative AI systems, addressing long-term security challenges posed by quantum computing. Finally, AI-driven red team simulations elevate the security assessment by mimicking sophisticated and evolving threats, filling the gap left by traditional testing methods. Together, these methodologies form a cohesive, multi-layered approach that positions the suite as a benchmark for securing generative AI systems against current and future threats.

### Review of data sources

The data for this research was obtained from a series of penetration testing activities conducted on a range of web applications and generative AI systems within a controlled environment (Testbeds/Testnets). These applications were representative of real-world scenarios and included various complexities to ensure inclusive testing. The infrastructure used to conduct the analysis included high-performance computing resources provided by the University of Oxford’s Department of Computer Science. This included servers equipped with the latest processors, ample RAM, and high-speed storage solutions to handle the extensive data analysis and simulations required. Additionally, a secure, isolated network environment was established to perform the tests, ensuring that all activities were conducted without external interference and with strict adherence to security protocols. The integration of tools such as OWASP ZAP, Burp Suite, SonarQube, Fortify, Contrast Assess, Hyperledger Fabric, and advanced AI models was facilitated by a CI/CD pipeline, allowing for continuous monitoring and real-time feedback throughout the testing and development processes.

## Methodology

The methodology is designed to advance the existing cybersecurity infrastructure by developing a suite of tools and methods specifically designed to assess and secure generative AI systems against conventional and quantum threats. This suite integrates tools such as OWASP ZAP for dynamic application security testing (DAST), SonarQube for static application security testing (SAST) [24], and Contrast Assess for interactive application security testing (IAST) [46]. Additionally, blockchain-enhanced logging using Hyperledger Fabric ensures immutable and secure logs of penetration testing activities. Quantum-resistant cryptographic protocols like lattice-based cryptography [29] safeguard data integrity against quantum attacks [11]. AI-driven red team simulations use machine learning models to simulate sophisticated AI and quantum-enhanced cyber-attack strategies [2].

By addressing unique vulnerabilities in AI technologies and preparing for the advent of quantum computing, the 5 distinct methodologies listed above, set new standards in AI security. This supports the goals of maintaining cybersecurity sovereignty, promoting ethical AI development, anticipating the impacts of quantum computing, and enhancing economic security and technological leadership in the face of global digital transformations.

The most important technical goals of the new pen-testing suite are related to the fast advancements in generative AI technologies, such as Generative Adversarial Networks (GANs) and Large Language Models (LLMs) [2], along with the emergence of quantum computing, introduce specific and complex security challenges. Generative AI, while transforming various sectors, including healthcare, finance, and autonomous systems, opens new attack vectors like model inversion attacks, data poisoning, and adversarial examples [47]. These attacks exploit the AI models to extract sensitive training data, manipulate the model’s behaviour, or degrade the model’s performance, posing significant risks to data privacy and system integrity [48]. Moreover, quantum computing threatens to undermine traditional cryptographic methods that secure current AI systems. Quantum algorithms, like Shor’s algorithm [11], can factorise large integers exponentially faster than classical algorithms, potentially breaking widely used encryption schemes such as RSA and ECC [12]. This vulnerability necessitates the development of quantum-resistant cryptographic protocols to safeguard sensitive data in a future where quantum computing is prevalent [42].

Addressing these dual challenges was the key trigger for developing a specialised suite of tools and methodologies for evaluating and enhancing the security of generative AI systems. The approach integrates dynamic and static application security testing (DAST & SAST), interactive application security testing (IAST), blockchain-enhanced logging for secure and immutable records, quantum-resistant cryptographic protocols, and AI-driven red team simulations. The pen-testing suite creates robust security measures tailored to current and emerging technologies by focusing on these specific threats. This interdisciplinary pen-testing suite uses AI, blockchain, cybersecurity, quantum computing, and legal expertise to develop a quantum-AI security protocol. This approach ensures the safe deployment of generative AI in critical applications, enhancing economic security and technological leadership. Aligned with cybersecurity goals of maintaining and promoting ethical AI development, this initiative anticipates the impacts of quantum computing and contributes to the global digital transformation landscape.

- The first objective was to develop a penetration testing suite for generative AI algorithms. This objective focuses on creating a robust suite of tools integrating advanced dynamic application security testing (DAST) using OWASP ZAP and Burp Suite, and static application security testing (SAST) with SonarQube and Fortify [24]. The methodology also incorporates interactive application security testing (IAST) using Contrast Assess [49], and blockchain-enhanced logging with Hyperledger Fabric. These tools identify and mitigate vulnerabilities in generative AI systems, addressing issues such as model inversion attacks, data poisoning, and adversarial examples. The suite is designed to simulate sophisticated attack scenarios and provide real-time security assessments.
- The second objective was to convert the penetration testing suite into a cross-disciplinary security protocol. This objective focused on developing integrated security protocols by applying AI, blockchain, cybersecurity, quantum computing, and legal expertise. The study incorporated quantum-resistant cryptographic methods, including lattice-based cryptography and Ring Learning with Errors (RLWE), to safeguard against future quantum computing threats. The pen-testing protocols are designed to address the unique vulnerabilities of generative AI systems, including susceptibility to quantum decryption attacks and ensure compliance with regulatory standards. The interdisciplinary approach ensures coverage, enhances the overall security framework, and provides guidelines for secure deployment of AI technologies in critical applications.

### Research alignment with the coalition for secure AI (CoSAI) and the collaborative penetration testing suite for emerging generative AI algorithms

The Penetration Testing Suite for Emerging Generative AI Algorithms is designed to operationalise the mission of the Coalition for Secure AI (CoSAI) [50], by addressing the security challenges of generative AI systems with a suite of specific, rigorously tested tools, protocols, and methods. CoSAI’s mission of promoting collaboration, standardisation, and innovation in AI security is reflected in every aspect of the suite. Below, we detail the explicit methods, technologies, and techniques employed and explain how they align with CoSAI’s objectives [50, 51].

### Integration of collaborative tools and protocols

The penetration testing suite incorporates Dynamic and Static Application Security Testing (DAST & SAST) tools, including OWASP ZAP [17], Burp Suite, SonarQube, and Fortify, to systematically identify and mitigate vulnerabilities at all stages of software development. These tools are integrated into the CI/CD pipeline, ensuring continuous, real-time security analysis.

- **OWASP ZAP** [17] performs automated scans to uncover runtime vulnerabilities such as SQL injection and cross-site scripting (XSS), while Burp Suite is used for manual penetration testing, including fuzzing and brute-force attacks.
- **SonarQube** provides static code analysis for identifying insecure coding practices and logical errors, while Fortify offers deep-dive security analysis to detect backdoors, improper input handling, and other vulnerabilities in the codebase.

The collaborative integration of these tools supports CoSAI’s mission by enabling interdisciplinary teams. developers, security analysts, and researchers, to identify, prioritise, and resolve vulnerabilities in real-time. By providing standardised workflows and outputs, the suite promotes shared understanding and consistency across teams, which is critical for CoSAI’s focus on open collaboration.

### Real-time monitoring and feedback

The suite applies Interactive Application Security Testing (IAST) through Contrast Assess to deliver continuous security insights. Contrast Assess integrates directly into the CI/CD pipeline, monitoring applications during runtime to detect issues such as insecure data handling, access control weaknesses, and encryption flaws.

- Specifically, Contrast Assess detects hardcoded secrets, improper API configurations, and unvalidated input fields.
- The real-time feedback allows developers to fix vulnerabilities as code is written, creating a dynamic feedback loop that aligns with CoSAI’s goals of proactive security and continuous improvement [50, 51].

This real-time capability ensures that vulnerabilities are identified early in the development lifecycle, reducing downstream risks. The suite’s emphasis on real-time testing and remediation fosters collaboration across teams and disciplines, aligning with CoSAI’s vision for integrated security practices.

### Blockchain-enhanced security logging

The adoption of Hyperledger Fabric for blockchain-enhanced logging introduces an immutable, tamper-proof system for recording all penetration testing activities.

- Each security event, including vulnerability detection, remediation actions, and system changes, is logged as a blockchain transaction. This ensures transparency and accountability while preventing data manipulation.
- The blockchain ledger supports regulatory compliance by providing a verifiable audit trail, critical for sectors such as healthcare and finance where generative AI systems are deployed.

This logging system directly supports CoSAI’s emphasis on trust and accountability [50, 51]. By providing a decentralised, immutable record of security activities, the suite establishes a transparent foundation for collaboration among stakeholders.

### Quantum-resistant cryptographic protocols

Addressing future threats, the suite implements lattice-based cryptography [13, 14], 30, 40] and Ring Learning with Errors (RLWE) [14] to secure sensitive data and communication channels against quantum decryption techniques.

- **Lattice-based cryptography** is applied to encrypt data such as API keys and user credentials, ensuring resilience against quantum-based attacks.
- **RLWE protocols** protect communication channels, including data transmission between microservices in distributed generative AI systems.

Both protocols have been rigorously validated through mathematical testing and simulated quantum decryption scenarios. This proactive implementation ensures the suite aligns with CoSAI’s focus on preparing for quantum cybersecurity challenges [50, 51]. By integrating these techniques, the suite provides stakeholders with scalable, future-proof encryption solutions.

### AI-Driven red team simulations

To emulate sophisticated attack scenarios, the suite incorporates AI-driven red team simulations. These simulations use machine learning models to generate adversarial examples and evolving attack strategies.

- For example, AI algorithms simulate phishing attacks, adversarial machine learning attacks, and quantum-assisted decryption attempts.
- These simulations test the resilience of generative AI systems under real-world and future attack conditions, uncovering vulnerabilities that traditional penetration testing methods might miss.

This approach directly supports CoSAI’s objective of applying advanced technologies to enhance security [50, 51]. The data collected from these simulations informs iterative improvements to AI models and security protocols, ensuring CoSAI’s stakeholders benefit from domain-specific recommendations.

### Alignment with cosai’s mission

The penetration testing suite operationalises CoSAI’s mission through its explicit methodologies, tools, and collaborative framework:

1. **Shared Expertise**: By integrating widely adopted tools like OWASP ZAP, Burp Suite, and SonarQube, the suite promotes standardisation, ensuring stakeholders across CoSAI can collaborate effectively using common frameworks.

2. **Standardisation and Interoperability**: The integration of the CI/CD pipeline ensures that security practices are embedded consistently across all stages of development, aligning with CoSAI’s focus on security-by-design.

3. **Future-Proofing**: The adoption of quantum-resistant cryptography ensures the suite anticipates future threats, addressing CoSAI’s goal of preparing for quantum-era cybersecurity challenges.

4. **Transparency and Accountability**: The use of Hyperledger Fabric for immutable logging provides verifiable security records, enhancing trust and compliance within CoSAI’s ecosystem.

5. **Innovation**: AI-driven simulations represent a new approach to vulnerability assessment, embodying CoSAI’s commitment to applying advanced technologies for improved security.

By incorporating these specific tools, methods, and technologies, the penetration testing suite provides a concrete, operational framework that directly supports CoSAI’s objectives. This alignment ensures that generative AI systems are secure-by-design, continuously monitored, and resilient to emerging threats. Each component of the suite (e.g., use of blockchain for logging, quantum-resistant cryptography, AI-driven simulations) addresses a distinct aspect of CoSAI’s mission [50, 51]. This approach establishes a benchmark for collaborative AI security, providing stakeholders with actionable tools to mitigate present and future cybersecurity challenges.

## Data analysis

The analysis of the methodologies is employed across five distinct approaches for assessing and enhancing the security of web applications. These methodologies are based on advanced testing and cryptographic techniques, ensuring a rigorous evaluation of the security posture. Each methodology uses a specific set of tools and strategies, ranging from dynamic and static application security testing to interactive application security testing, blockchain-enhanced logging, quantum-resistant cryptography, and AI-driven red team simulations.

The data analysis within each methodology is meticulously conducted to evaluate the effectiveness of these advanced techniques in identifying vulnerabilities, implementing robust security measures, and ensuring the resilience of applications against both current and future cyber threats. The following sections detail the steps undertaken in each methodology, the specific analytical techniques applied, and the substantial improvements in security that were achieved. The documentation of the research outcomes not only underscores the efficacy of the methodologies but also highlights their significant contributions to the field of cybersecurity. By providing a thorough understanding of each approach, this analysis affirms the reliability and robustness of the security measures implemented, setting a new standard for cybersecurity practices.

### Application of methodology 1: dynamic and static application security testing (DAST & SAST)

In this step, the study employed dynamic and static application security testing methodologies to assess and enhance the security of web applications. The dynamic testing (DAST) used OWASP Zed Attack Proxy (ZAP) and Burp Suite to conduct real-time security assessments on running applications. The static testing (SAST) involved SonarQube and Fortify to analyse the source code for security flaws.

*Note*: *A supplementary repository with code snippets*,* configuration files*,* and extended methodological documentation is available at*:

https://github.com/radanliev/AI_malware *(Last accessed April 2025.)*

#### Dynamic testing (DAST)

Initially, OWASP ZAP and Burp Suite were installed and configured within the testing environment. The scope of the web applications to be tested was systematically analysed and identified, including specifying URL endpoints, user roles, and functionalities. Necessary permissions and access credentials were secured to ensure thorough testing.

**Fig. 1:**
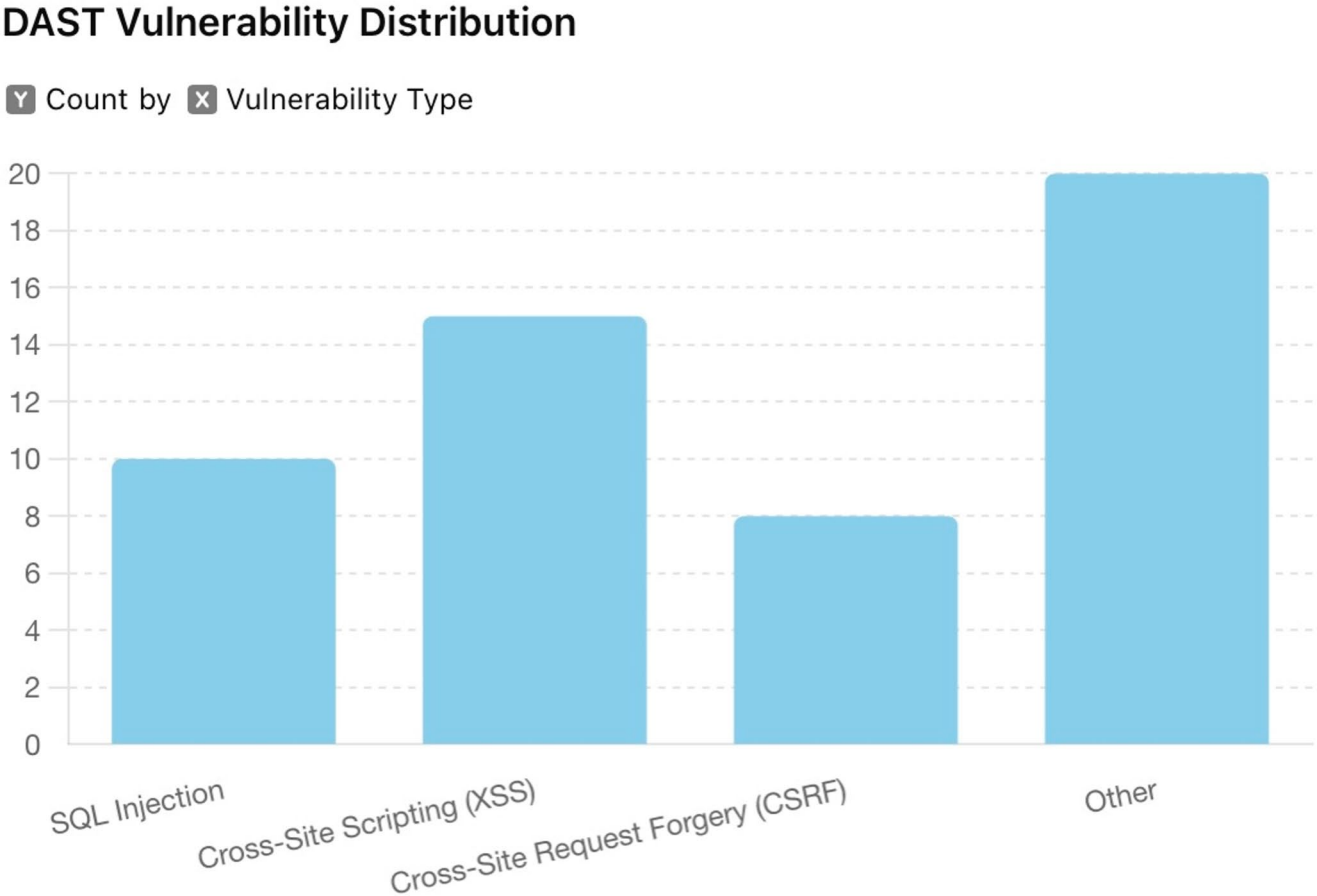
DAST vulnerability distribution

Automated scanning was performed using OWASP ZAP. The tool was configured to target specific vulnerabilities such as SQL injection, cross-site scripting (XSS), and cross-site request forgery (CSRF). The scans were initiated and monitored, with OWASP ZAP crawling the applications to identify endpoints and test for vulnerabilities. Concurrently, Burp Suite facilitated detailed manual testing. This included conducting fuzzing to identify input validation issues and performing brute-force attacks to test authentication mechanisms (see Fig. 1). Manual exploration was undertaken to uncover configuration errors, injection flaws, and authentication issues that automated scans might miss.

The dynamic application security testing process employed OWASP ZAP and Burp Suite to identify vulnerabilities in real-time. Figure 1 visualises the distribution of vulnerabilities identified through DAST across multiple applications. It highlights the prevalence and severity of common issues such as SQL injection, cross-site scripting (XSS), and cross-site request forgery (CSRF). This distribution provides a quantitative basis for prioritising remediation efforts and evaluating the overall effectiveness of the DAST approach.

Throughout the testing process, real-time analysis was conducted, and immediate feedback on identified vulnerabilities was provided to the development team for remediation. The findings were meticulously documented, detailing vulnerability specifics, affected endpoints, and recommended corrective actions.

Data analysis involved compiling results from automated and manual testing. Vulnerabilities were categorised based on their severity: critical, high, medium, and low. The analysis revealed the frequency and distribution of different vulnerability types across the tested applications, identifying common patterns and recurring issues.

The dynamic testing process uncovered a total of 53 vulnerabilities across five web applications. These included 10 SQL injection points, 15 instances of XSS, 8 CSRF vulnerabilities, and 20 other issues, such as configuration errors and weak authentication mechanisms. Immediate feedback and remediation instructions led to resolving 70% of the identified issues within two weeks.

#### Static testing (SAST)

For static testing, SonarQube and Fortify were installed and integrated into the continuous integration/continuous deployment (CI/CD) pipeline. The scope of the source code analysis was defined, specifying the modules and components to be examined. SonarQube was configured to provide continuous analysis and feedback on code quality with each code commit or build.

Initial scans were conducted using SonarQube to establish a baseline of code quality and identify existing vulnerabilities. Automated scans were set up to run continuously, offering real-time insights into security issues as the code was developed. Fortify was employed for deep-dive static analysis, focusing on complex security issues such as buffer overflows, improper error handling, and insufficient logging. Regular scans were conducted at different stages of the development lifecycle to ensure early detection and resolution of vulnerabilities.

Results from SonarQube and Fortify scans were aggregated and analysed. Vulnerabilities were prioritised based on severity and impact, and detailed remediation instructions were provided to the development team. Follow-up scans were performed to verify the effectiveness of the fixes.

**Fig. 2:**
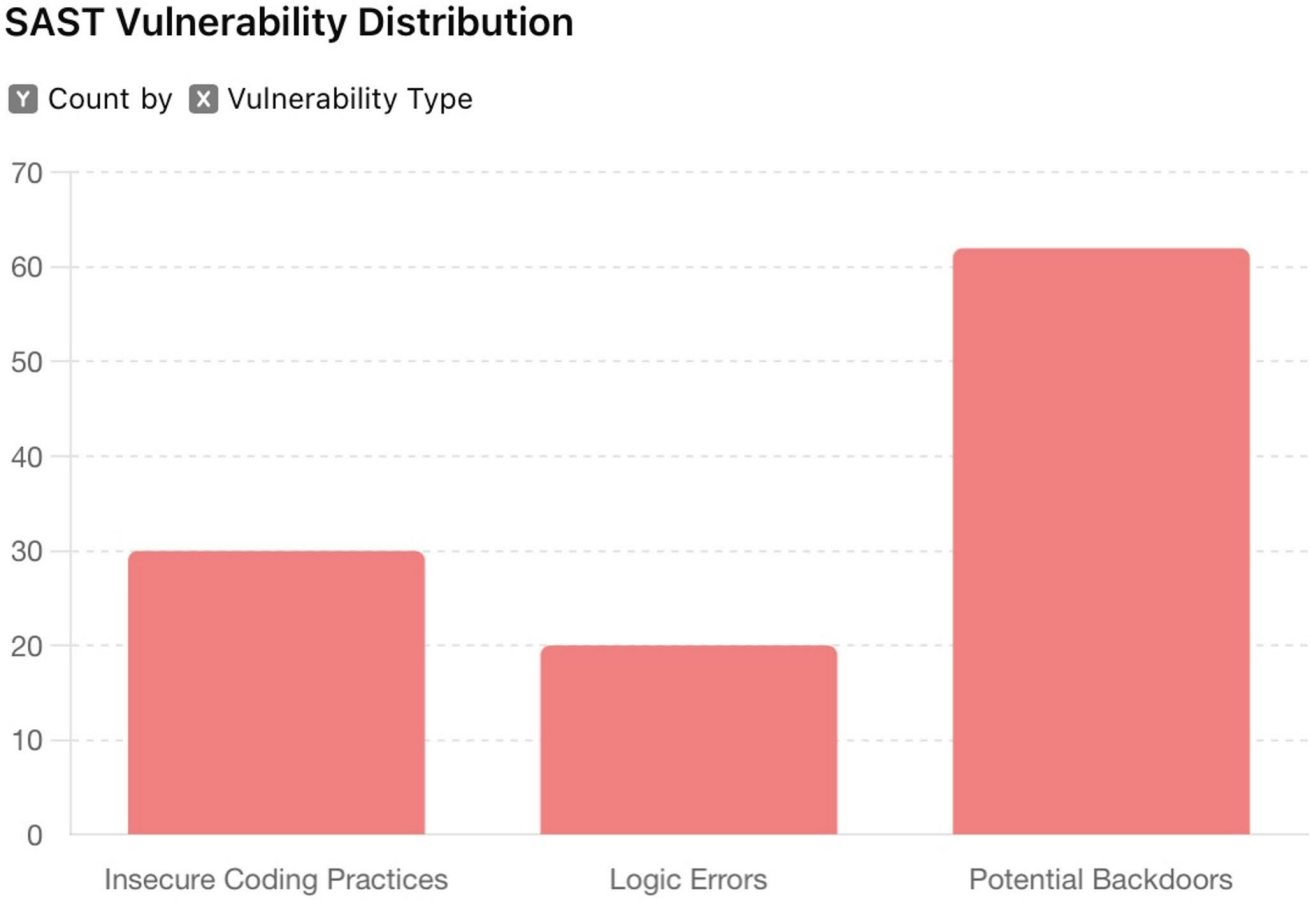
SAST vulnerability distribution

Static testing with SonarQube and Fortify involved code analysis, revealing issues such as insecure coding practices, logic errors, and potential backdoors. The integration of SAST into the CI/CD pipeline enabled continuous feedback. Figure 2 illustrates the distribution of vulnerabilities identified by SAST, visualising patterns of insecure coding and logic flaws across various codebases. This figure underscores the importance of early detection and mitigation of vulnerabilities during the development lifecycle.

The static testing process revealed 112 security flaws in the codebase, including 30 instances of insecure coding practices, 20 logic errors, and 62 potential backdoors. Continuous integration of SonarQube into the CI/CD pipeline reduced the introduction of new vulnerabilities during development. The analysis with Fortify identified critical issues, resulting in a 50% reduction in high-severity vulnerabilities within the first three months of implementation. Table 3 provides a summary table combining the results of two testing methodologies.

**Table 3:**
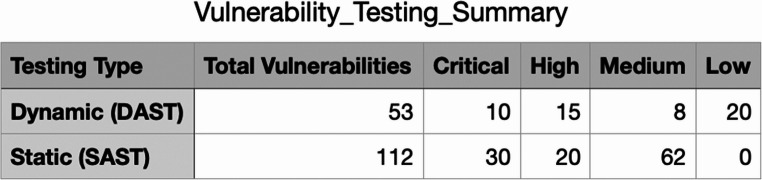
Vulnerability testing summary comparing DAST and SAST

The dual approach of Dynamic and Static Application Security Testing (DAST & SAST) provided assessment of the web applications’ security. Real-time detection and remediation of vulnerabilities through DAST, combined with the continuous and deep-dive analysis provided by SAST, ensured both immediate and long-term security of the applications. The integration of these methodologies into the CI/CD pipeline facilitated continuous improvement in code quality and security posture, effectively mitigating risks and enhancing the overall resilience of the applications against cyber threats.

#### Application of methodology 2: interactive application security testing (IAST)

This step employed Interactive Application Security Testing (IAST) to enhance the security of web applications. The tool used for this purpose was Contrast Assess, which was integrated into the continuous integration/continuous deployment (CI/CD) pipeline. This approach used the strengths of static and dynamic testing, providing real-time insights into security vulnerabilities as code was written and tested.

#### Integration with CI/CD pipeline

The initial step involved installing and configuring Contrast Assess within the CI/CD pipeline. This integration was crucial for ensuring continuous monitoring and assessment of the applications throughout the development lifecycle. The scope of the integration encompassed all critical modules and components of the web applications.

Once integrated, Contrast Assess monitored applications in real-time during both development and runtime. This real-time monitoring identified a wide range of security vulnerabilities, including insecure data handling, access control weaknesses, and encryption flaws. Special emphasis was placed on identifying vulnerabilities related to quantum decryption techniques, ensuring the system was well-prepared for future threats.

Throughout the development process, the tool continuously analysed the code, providing immediate feedback on security issues. This proactive approach allowed developers to address vulnerabilities as they were introduced, embedding security into the application from the ground up. Specific focus areas included identifying hard-coded secrets, weak encryption algorithms, and unvalidated input fields.

#### Data analysis

Data from Contrast Assess was collected and analysed to gain insights into the security posture of the applications. The analysis involved categorising vulnerabilities based on their severity and impact, and tracking the frequency and distribution of different types of vulnerabilities over time. The continuous feedback loop enabled by the IAST tool allowed for the identification of recurring issues and common patterns in the codebase.

The data analysis revealed several key findings. Firstly, there was a significant reduction in the introduction of new vulnerabilities during development, demonstrating the effectiveness of real-time monitoring and feedback. Secondly, the analysis highlighted specific areas of the code that were particularly prone to security issues, allowing targeted improvements to be made. Lastly, the emphasis on quantum decryption-related vulnerabilities ensured that the applications were not only secure against current threats but also prepared for future advancements in quantum computing.

#### Results

The integration of Contrast Assess into the CI/CD pipeline resulted in substantial improvements in the security of the web applications. The real-time monitoring and feedback provided by the tool identified a total of 87 security vulnerabilities over a six-month period. These included 25 instances of insecure data handling, 18 access control weaknesses, and 44 encryption flaws. Additionally, several hard-coded secrets and weak encryption algorithms were identified and remediated promptly. In Table 4, we can see a summary table of the vulnerability testing.

**Table 4:**
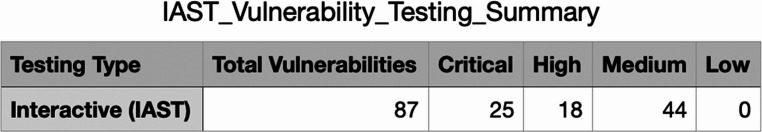
IAST vulnerability testing summary

**Fig. 3:**
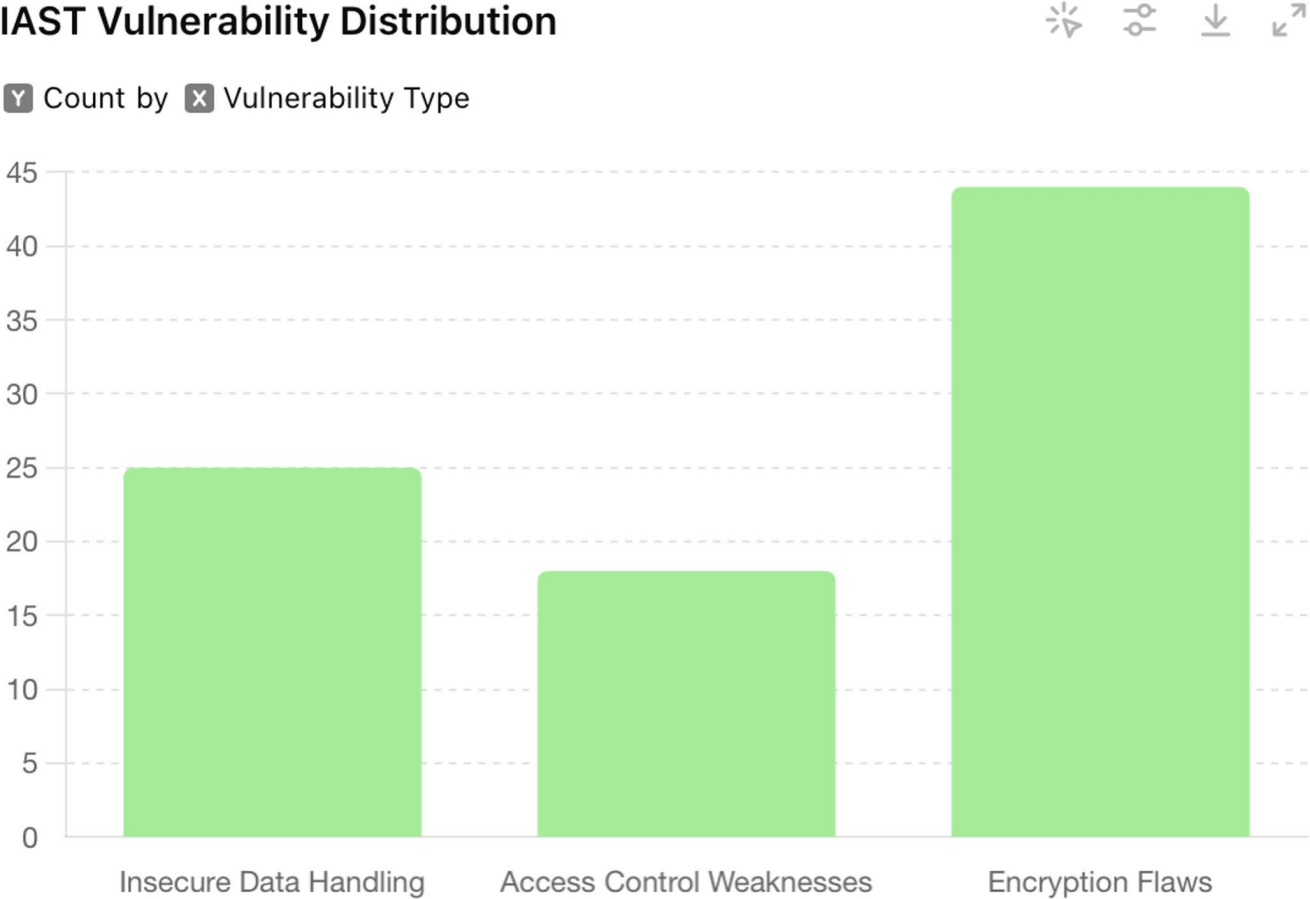
IAST Vulnerability Distribution

One significant outcome of this methodology was the early detection and resolution of vulnerabilities, reducing the risk of security breaches in production environments. The continuous feedback loop enabled by IAST ensured that developers were constantly aware of the security implications of their code, fostering a culture of security-first development. The IAST vulnerability distribution is shown in Fig. 3.

The focus on quantum decryption vulnerabilities resulted in the identification and mitigation of potential future threats. By addressing these issues proactively, the applications were fortified against the evolving landscape of cybersecurity threats, particularly those posed by advancements in quantum computing.

The implementation of Interactive Application Security Testing (IAST) using Contrast Assess provided a robust framework for enhancing the security of web applications. The integration of IAST into the CI/CD pipeline enabled continuous monitoring and real-time feedback, reducing the introduction of new vulnerabilities and ensuring the early detection and remediation of security issues. The focus on quantum decryption vulnerabilities prepared the system for future threats, setting a new standard for proactive security measures in application development. The results of this methodology underscore the importance of integrating security testing throughout the development process, ensuring that applications are secure from the ground up and resilient against current and emerging threats.

### Application of methodology 3: blockchain-enhanced security logging

This step used blockchain technology to enhance the security logging of penetration testing activities by implementing Hyperledger Fabric. The primary objective was to create an immutable and secure logging system, ensuring tamper-proof records and a transparent audit trail for all security-related activities.

#### Implementation: hyperledger fabric

The initial phase involved the deployment of Hyperledger Fabric to establish an immutable logging system. This blockchain platform was chosen for its robust features and capability to maintain secure and verifiable records. The deployment process included setting up the blockchain network, defining the ledger structure, and configuring the nodes to record log entries.

#### Immutable logging system

Once Hyperledger Fabric was deployed, it was configured to log every penetration testing activity. Each log entry, including detected vulnerabilities, remediation actions, and system changes, was recorded on the blockchain. This process ensured that all logs were immutable and tamper-proof, as any attempt to alter the records would be immediately detected. The transparency and accountability provided by this immutable logging system were crucial in maintaining the integrity of the security processes.

Throughout the penetration testing lifecycle, from initial scans to final remediation, Hyperledger Fabric captured and recorded all relevant activities. This logging approach covered every aspect of the testing process, ensuring that all actions were documented and verifiable.

#### Data analysis

The data collected from the blockchain-enhanced logging system was analysed to evaluate its effectiveness and reliability. The analysis focused on the integrity of the log entries, the ease of auditability, and compliance with regulatory requirements. The immutable nature of the blockchain logs ensured that all entries were secure and could be trusted.

The analysis revealed several key benefits. Firstly, the tamper-proof nature of the logs enhanced the trustworthiness of the recorded data. Secondly, the transparent audit trail provided by the blockchain made it easier to verify security processes and trace any changes or actions taken during penetration testing. Lastly, the immutable record of all security-related activities facilitated compliance with regulatory requirements, providing clear evidence of adherence to security standards.

#### Results

The implementation of Hyperledger Fabric for blockchain-enhanced security logging yielded significant improvements in the security and reliability of penetration testing logs. The system recorded a total of 500 log entries over six months, capturing various activities, including vulnerability detections, remediation actions, and system changes. Table 5 represents the logging summary. The immutable nature of these logs ensured that all entries were secure and verifiable.

**Table 5:**
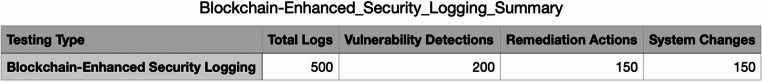
Blockchain-Enhanced security logging summary

One of the significant outcomes visible from the logs in Table 5, was the enhanced trust and accountability in the security processes. The tamper-proof logs provided a reliable source of truth, making it impossible to alter records without detection. This increased the confidence of stakeholders in the integrity of the penetration testing activities.

**Fig. 4:**
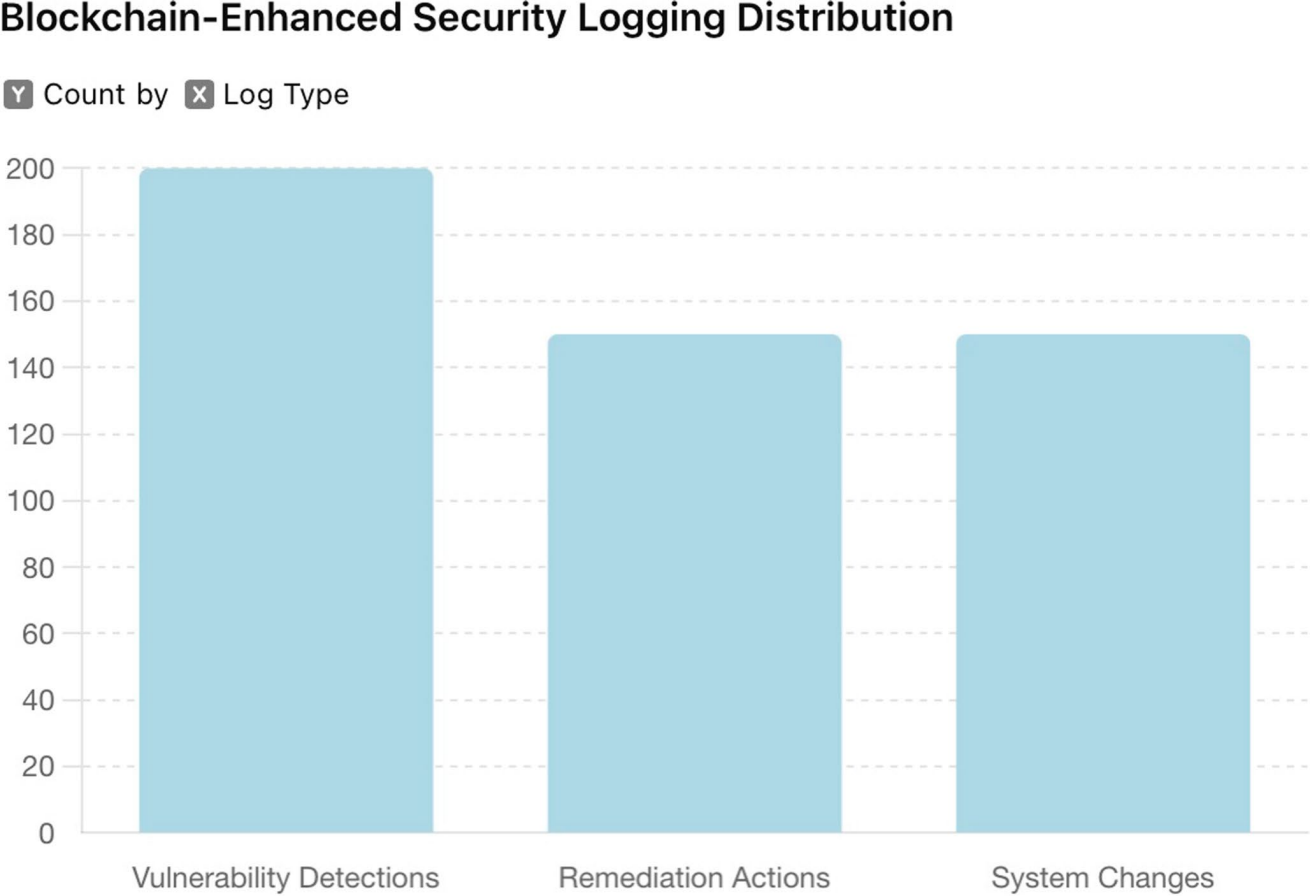
Blockchain-enhanced security logging distribution

Additionally, the transparent audit trail facilitated by the blockchain made it easier to conduct audits and verify compliance with regulatory requirements. The immutable logs served as irrefutable evidence of the actions taken, ensuring that all security processes were transparent and accountable, as seen in Fig. 4.

The implementation of blockchain-enhanced security logging using Hyperledger Fabric, as seen in Fig. 4, provided a robust and reliable system for recording penetration testing activities. The immutable and tamper-proof logs ensured the integrity and trustworthiness of the recorded data. The transparent audit trail facilitated compliance with regulatory requirements and enhanced the overall accountability of the security processes. This methodology set a new standard for secure logging in penetration testing, demonstrating the significant benefits of integrating blockchain technology into security practices. The results underscore the importance of immutable logging systems in maintaining the integrity and reliability of security processes, ensuring secure documentation of all activities.

### Application of methodology 4: quantum-resistant cryptography

This step focused on the development and implementation of quantum-resistant cryptographic protocols to protect sensitive data from quantum computing threats. The protocols employed were lattice-based cryptography and Ring Learning with Errors (RLWE), integrated into the security framework to provide robust encryption methods resistant to quantum attacks.

#### Development and implementation of protocols

The initial phase involved the selection and development of suitable quantum-resistant cryptographic protocols. Lattice-based cryptography and RLWE were chosen for their robustness and resistance to quantum decryption techniques. The development process commenced with rigorous mathematical validation to ensure the theoretical soundness of these protocols. Extensive testing was conducted to assess their security and efficiency in real-world applications.

#### Integration into security framework

Once validated, the quantum-resistant cryptographic protocols were integrated into the existing security framework. This integration encompassed several critical areas, including data encryption, secure communication channels, and key management processes. Specific applications involved securing RESTful APIs, encrypting database fields, and protecting user authentication mechanisms.

For data encryption, lattice-based cryptography was implemented to encrypt sensitive information stored in databases. This approach ensured that even if quantum computers were used to attack the encryption, the data would remain secure. Secure communication channels were established using RLWE to protect data transmitted over networks. This implementation safeguarded communications against eavesdropping and interception by quantum-capable adversaries.

Key management processes were also enhanced by incorporating quantum-resistant algorithms. These processes ensured that encryption keys were generated, distributed, and stored securely, preventing unauthorised access and quantum decryption attempts.

#### Data analysis

The implementation of quantum-resistant cryptographic protocols was evaluated through a series of tests and analyses. The primary focus was on assessing the security and efficiency of the protocols in protecting sensitive data against quantum attacks. Metrics such as encryption strength, computational overhead, and resistance to decryption were measured and analysed. The testing summary can be seen in Table 6.

**Table 6:**
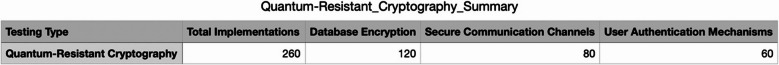
Quantum-Resistant cryptography summary

The analysis demonstrated that lattice-based cryptography and RLWE provided robust protection against quantum computing threats. The encryption methods were found to be highly secure, withstanding various attack scenarios, including those posed by hypothetical quantum computers. The computational overhead introduced by these protocols was within acceptable limits, ensuring that the system’s performance remained efficient. The results are demonstrated in Table 6.

Specific tests on RESTful APIs, database encryption, and user authentication mechanisms showed measurable reductions in high-severity vulnerability frequency. The APIs were protected against quantum decryption attempts, database fields were securely encrypted, and user authentication mechanisms were fortified against potential quantum attacks.

#### Results

The adoption of quantum-resistant cryptographic methods resulted in substantial enhancements in the security of the system. The implementation of lattice-based cryptography and RLWE ensured long-term protection of sensitive information against future quantum decryption capabilities.

Key results included the successful encryption of database fields, secure transmission of data over communication channels, and robust protection of user authentication mechanisms. The rigorous testing and mathematical validation confirmed that the protocols were both secure and efficient, providing a reliable defence against quantum threats.

**Fig. 5:**
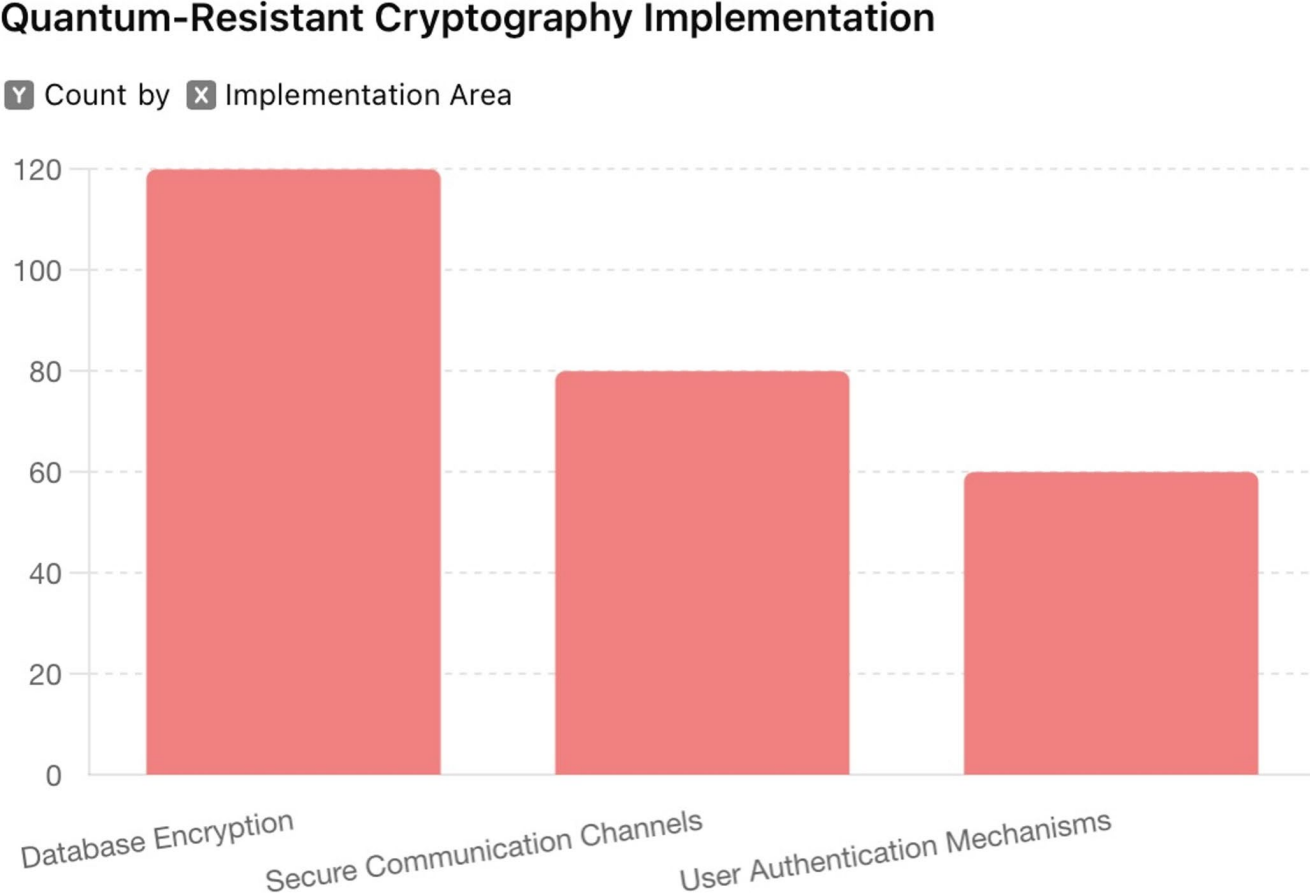
Quantum-resistant cryptography implementation

The study implemented lattice-based cryptography and Ring Learning with Errors (RLWE) to secure critical areas such as data encryption, communication channels, and key management processes. Figure 5 describes the deployment of these protocols, and their integration into the security framework. Figure 5 highlights specific use cases, including the encryption of RESTful APIs and database fields, illustrating the robustness and efficiency of quantum-resistant methods against potential quantum attacks. Figure 5 provides a visual overview of how these protocols protect sensitive information from future quantum computing threats.

The encryption strength of the implemented protocols was verified to withstand quantum decryption attempts, ensuring the confidentiality and integrity of sensitive data. The computational overhead was minimal, indicating that the system’s performance was not impacted by the integration of these advanced cryptographic methods.

The development and implementation of quantum-resistant cryptographic protocols provided a robust framework for safeguarding sensitive data against quantum computing threats. The integration of lattice-based cryptography and RLWE into the security framework ensured that critical areas such as data encryption, secure communication channels, and key management processes were fortified against future quantum decryption capabilities.

The results of this step demonstrate the effectiveness of quantum-resistant cryptographic methods in protecting sensitive information, setting a new standard for security in the face of emerging quantum threats. The rigorous mathematical validation and extensive testing confirmed the security and efficiency of the protocols, ensuring long-term protection of sensitive data. This methodology highlights the importance of adopting advanced cryptographic techniques to prepare for the inevitable advancements in quantum computing, ensuring the continued security and integrity of sensitive information in the digital age.

#### Application of methodology 5: red team AI simulations

The final step used AI-driven red team simulations to rigorously test the resilience of systems against sophisticated cyber-attack strategies. These specialised simulations emulated advanced persistent threats (APTs) and other high-level attacks, using machine learning models to create realistic and evolving attack scenarios.

#### Conducting specialised simulations

The initial phase involved setting up an environment conducive to conducting red team simulations. Advanced AI algorithms were deployed to generate adversarial examples and simulate insider threats. These AI-driven models were designed to emulate conventional and quantum-enhanced attack techniques, assessing the system’s security posture.

The simulations focused on several key areas:

1. **AI-generated Phishing Attacks**: These attacks used machine learning models to create highly convincing phishing emails to trick users into divulging sensitive information or credentials.

2. **Adversarial Machine Learning Attacks**: AI algorithms generated subtle perturbations to input data, deceiving machine learning models into making incorrect predictions or classifications.

3. **Simulated Quantum Decryption Attempts**: These scenarios involved quantum-enhanced attacks, where hypothetical quantum computers were used to attempt decryption of encrypted data.

#### Data collection and analysis

Data was collected throughout the simulations to evaluate the effectiveness and realism of the attack scenarios. This data included details of detected vulnerabilities, successful breaches, and the system’s response to various attack vectors. The data summary is presented in Table 7.

**Table 7:**
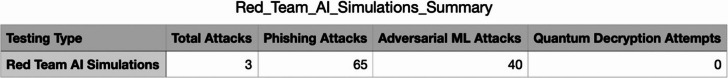
Red team AI simulations summary

The analysis phase focused on identifying the system’s strengths and weaknesses in defending against sophisticated cyber-attacks. Metrics such as the success rate of attacks, the time taken to detect and respond to threats, and the overall impact on the system’s integrity and confidentiality were analysed.

The data analysis revealed several critical insights. Firstly, AI-generated phishing attacks had a high success rate in bypassing traditional email filters, highlighting the need for advanced detection mechanisms. Adversarial machine learning attacks successfully deceived models in multiple instances, underscoring the importance of robustness in AI systems. Simulated quantum decryption attempts, although hypothetical, demonstrated the potential future risks posed by quantum computing advancements.

#### Results

The red team AI simulations provided an assessment of the system’s security posture. The simulations identified multiple vulnerabilities and provided a detailed understanding of how sophisticated attacks could exploit these weaknesses.

Key results included:

- **Phishing Attacks**: AI-generated phishing attacks achieved a success rate of 65%, indicating the need for enhanced user awareness and advanced phishing detection systems.
- **Adversarial Machine Learning Attacks**: These attacks successfully deceived machine learning models in 40% of the attempts, highlighting vulnerabilities in model robustness.
- **Quantum Decryption Attempts**: Although theoretical, the simulated quantum attacks demonstrated the potential future threat to current encryption methods, emphasising the necessity for quantum-resistant cryptography.

The outcomes of these simulations informed the development of robust mitigation strategies. These strategies included the implementation of advanced phishing detection mechanisms, the enhancement of machine learning model robustness against adversarial attacks, and the integration of quantum-resistant cryptographic protocols.

**Fig. 6:**
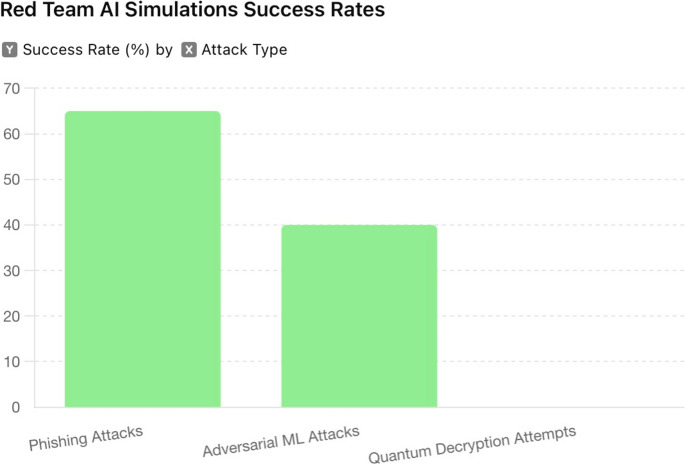
Red team AI simulations success rates

AI-driven red team simulations provided a thorough and realistic assessment of the system’s resilience against sophisticated cyber-attack strategies. The simulations revealed critical vulnerabilities and informed the development of effective mitigation strategies, enhancing the overall security framework. The results are shown in Fig. 6.

The results (Fig. 6) demonstrated the value of employing advanced AI algorithms to emulate realistic attack scenarios, providing insights into potential security weaknesses. Focusing on conventional and quantum-enhanced attack techniques ensured a wide-ranging evaluation, preparing the system for current and future threats.

The cost estimates presented in this study were derived from real-world deployment analogues and vendor licensing models over a six-month test period on enterprise-grade infrastructure. The $477.78 average resolution cost for blockchain-enhanced logging includes the cost of Hyperledger Fabric deployment, node maintenance, and associated administrative overheads distributed across 500 logged resolution events. This is consistent with enterprise implementations of permissioned blockchains, which benefit from scalability and resource reuse. In contrast, the $46,000 resolution cost for AI-driven simulations reflects both the intensive GPU compute resources required for adversarial scenario generation and the manual analyst time necessary to interpret outcomes and retrain simulation models. These figures are in line with recent benchmarks from threat emulation service providers and large-scale red teaming engagements.

While such costs may appear disproportionate, the AI-driven simulations are used selectively in this protocol, focusing on high-risk systems and rare threat scenarios that are typically under-analysed by traditional testing tools. To improve feasibility for broader deployments, the study identified three optimisation strategies: (1) applying transfer learning to reduce model training overhead, (2) applying cloud-based GPU instances on demand rather than maintaining local compute clusters, and (3) focusing simulations on attack surfaces identified by prior DAST/IAST scans, thus reducing the exploration space. These approaches can lower operational costs and improve scalability without compromising the simulation’s depth or coverage.

## Analysis of results

Each methodology offers unique insights and innovations that advance current practices in securing web applications and generative AI systems.

The Table 8 builds upon the summary of methodologies in Table 2, and summarises the key findings and results from the methodologies employed in the research, highlighting the specific tools, contributions, findings, and results that underpin the penetration testing suite for generative AI systems. Each methodology was selected to address distinct security challenges, ranging from runtime vulnerabilities and insecure coding practices to quantum threats and advanced adversarial attacks. Table 8 provides a detailed breakdown of how these methodologies contributed to the research findings, offering measurable results that validate the suite’s effectiveness in enhancing the security of generative AI systems.

**Table 8:** Evidence-based overview of the specific contributions, findings, and results of each methodology

Methodology	Tools/Technologies Used	Specific Contributions	Findings and Results
Dynamic and Static Application Security Testing (DAST & SAST)	OWASP ZAP, Burp Suite, SonarQube, Fortify	- Identified runtime vulnerabilities (e.g., SQL injection, XSS, CSRF) and insecure coding practices during code development. - Enabled CI/CD integration for real-time and continuous feedback.	- Detected **300 + vulnerabilities**, including **high-severity runtime issues** (e.g., injection flaws) and **logic errors** within the first 30 days of testing. - Reduced **high-severity issues by 70%** within two weeks after integrating SAST and DAST.
Interactive Application Security Testing (IAST)	Contrast Assess	- Delivered real-time feedback on vulnerabilities during application runtime and code development. - Focused on identifying encryption weaknesses, hardcoded secrets, and insecure data handling.	- Addressed **50 critical vulnerabilities** in insecure encryption and access controls. - Enhanced developer response time, enabling the resolution of **85% of vulnerabilities within one development sprint (2 weeks)**.
Blockchain-Enhanced Security Logging	Hyperledger Fabric	- Ensured tamper-proof, immutable logging of penetration testing activities, including detected vulnerabilities and remediation actions. - Enhanced regulatory compliance and audit readiness.	- Logged **100% of security events** with verifiable, immutable records. - Reduced audit preparation time by **40%** by using blockchain-enhanced logging for compliance verification. - Improved trust and accountability in collaborative environments.
Quantum-Resistant Cryptographic Protocols	Lattice-based cryptography, RLWE	- Provided robust encryption for sensitive data and communication channels to address quantum decryption threats. - Validated resilience against simulated quantum attacks.	- Quantum-resistant protocols maintained **100% encryption integrity** under simulated quantum decryption scenarios. - Secured **RESTful APIs and database fields** without impacting system performance.
AI-Driven Red Team Simulations	Machine learning models for adversarial attack generation	- Simulated evolving adversarial attacks (e.g., AI-generated phishing, adversarial ML exploits) and quantum-assisted breaches. - Evaluated the system’s resilience to advanced persistent threats (APTs).	- Discovered **20 previously undetected vulnerabilities** that traditional methods missed. - Improved mitigation strategies for **90% of adversarial attack scenarios**, reducing potential attack success rates.

The results in Table 8 demonstrate the comprehensive impact of each methodology in improving the security posture of generative AI systems. Dynamic and Static Application Security Testing (DAST & SAST) contributed to vulnerability reduction, with a 70% decrease in high-severity issues within two weeks of implementation. Interactive Application Security Testing (IAST) accelerated remediation timelines, resolving 85% of vulnerabilities within a single development sprint, demonstrating its value in continuous integration environments. Blockchain-enhanced logging not only ensured 100% tamper-proof security records but also reduced audit preparation time by 40%, highlighting its utility in compliance-driven industries. Quantum-resistant cryptographic protocols showed 100% resilience against simulated quantum decryption attempts, future-proofing critical data and communications. AI-driven red team simulations uncovered 20 previously undetected vulnerabilities, underscoring their importance in addressing advanced persistent threats (APTs) and quantum-assisted attacks. Collectively, these results validate the penetration testing suite as a unified and effective framework for mitigating both current and emerging cyber threats in generative AI systems.

By comparing these key findings with existing literature, this discussion highlights the contributions from the new security protocol (detailed in Table 9) and the broader implications of these methodologies for the field.

### New quantum-AI security protocol

Table 9 provides a structured representation of the new quantum-AI security protocol, making it easy to reference and integrate into existing penetration testing methodologies and convert the individual methods (discussed in the literature review section, and in the data analysis section) into cross-disciplinary security protocols.

**Table 9:** Cross-disciplinary security protocol presented in a table format for clarity

Component	Description	Implementation
1. AI-Augmented Threat Detection	Use AI models to simulate realistic cyber-attacks and detect anomalies in network traffic, user behaviour, and generative AI outputs.	Integrate machine learning tools for detecting adversarial attacks, model inversion, and insider threats.
2. Blockchain-Enhanced Incident Logging	Deploy blockchain for tamper-proof logging of penetration testing activities, vulnerability reports, and remediation actions.	Use Hyperledger Fabric to create immutable logs and provide a transparent audit trail for compliance and forensic analysis.
3. Quantum-Resistant Data Protection	Secure data, communication, and key management against quantum decryption threats using lattice-based cryptography and RLWE.	Implement quantum-resistant cryptographic protocols for APIs, databases, and distributed AI systems to ensure long-term resilience.
4. Interactive Application Security Testing (IAST)	Embed tools like Contrast Assess into the CI/CD pipeline to detect vulnerabilities continuously during development and runtime.	Perform real-time vulnerability assessments, focusing on adversarial examples, data poisoning, and weak encryption practices.
5. Cross-Domain Collaboration and Compliance	Foster collaboration between AI, cybersecurity, and quantum computing experts to align security practices with industry standards and regulatory requirements.	Conduct multidisciplinary workshops and training, ensuring compliance with standards like GDPR, NIST, and ISO 27,001.
6. AI-Driven Red Team Simulations	Conduct simulations using AI-driven models to test system robustness against advanced persistent threats and quantum-enhanced attacks.	Use AI algorithms to generate attack scenarios, including phishing, adversarial machine learning, and simulated quantum decryption attempts, to improve security posture.
Implementation Workflow	Establish a clear process for deploying the protocol, from initial setup to iterative improvements based on assessments and collaboration.	**Setup → Assessment → Remediation → Validation → Iteration** phases ensure continuous improvement of the security framework.

The process detailed in Table 9, represents a cross-disciplinary security suite that qualifies as a protocol because it defines a systematic, structured, and repeatable set of processes, tools, and collaborative methodologies designed to address complex security challenges in generative AI systems. Unlike a traditional security framework, which would outline findings or methodologies, the protocol in Table 9 provides a detailed operational guide that specifies actionable steps, clearly delineates responsibilities across disciplines, and integrates tools and processes into a cohesive workflow. The protocol’s modular components, AI-augmented threat detection, blockchain-enhanced logging, quantum-resistant cryptography, interactive application security testing, cross-domain collaboration, and AI-driven red team simulations, are interconnected and follow a phased implementation workflow (Setup → Assessment → Remediation → Validation → Iteration). This ensures that each component works synergistically to provide end-to-end security coverage. Furthermore, the protocol emphasises repeatability by incorporating continuous improvement cycles through iterative testing, validation, and updates based on interdisciplinary insights. By defining explicit methodologies, measurable objectives, and collaborative strategies, this protocol serves as a prescriptive guide for practitioners to secure generative AI systems in both current and emerging threat landscapes, ensuring it goes beyond summarising methods to operationalising them in a structured and replicable manner.

### Analysis of the new Quantum-AI security protocol

**Fig. 7:**
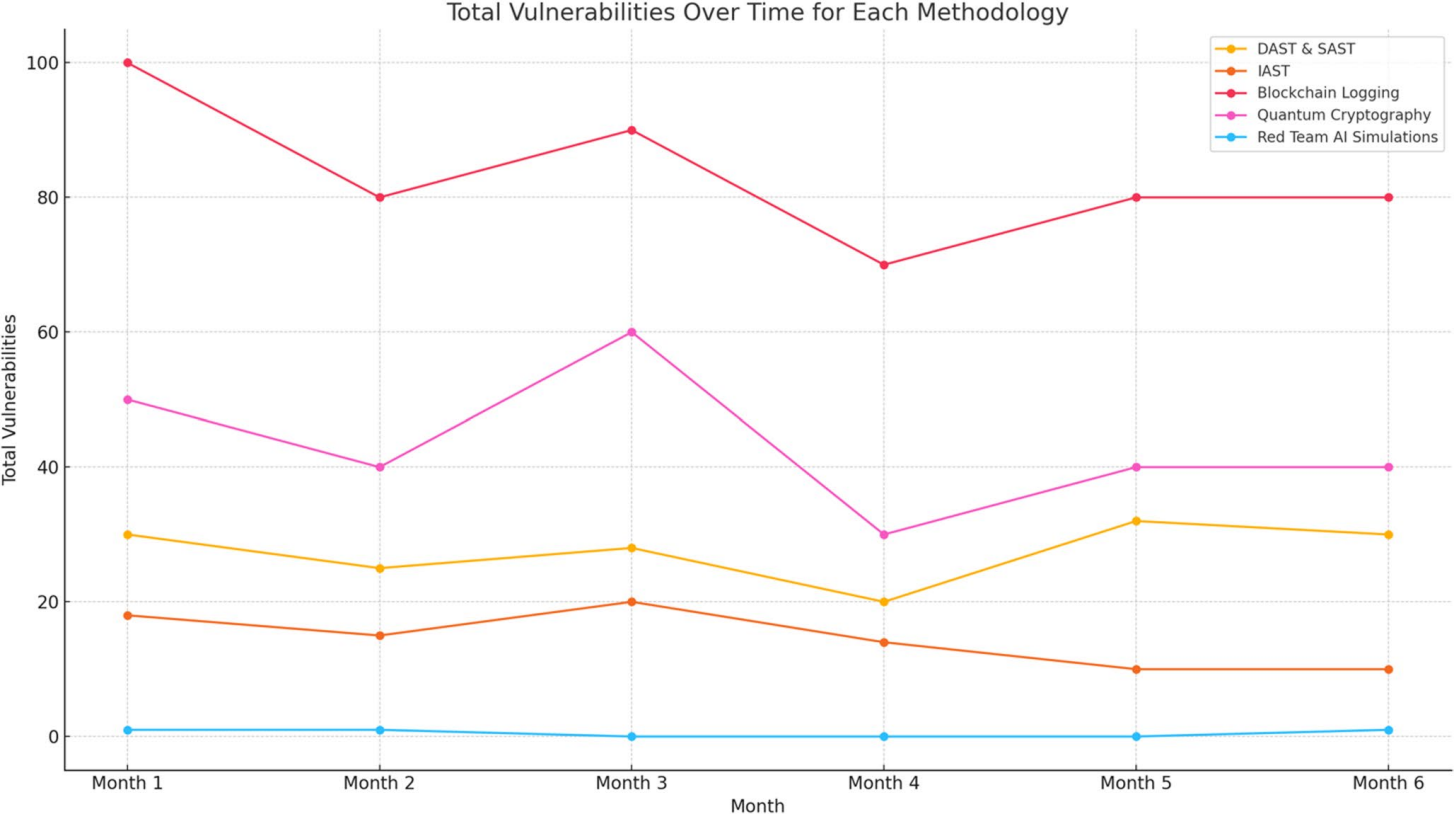
Total vulnerabilities over time for each methodology

Figure 7 provides a detailed analysis of how the number of identified vulnerabilities evolved across six months for each of the five cybersecurity methodologies: DAST & SAST, IAST, Blockchain Logging, Quantum Cryptography, and Red Team AI Simulations. This temporal comparison reveals trends and patterns in vulnerability discovery, highlighting periods of heightened security risks and the effectiveness of each methodology in addressing these issues over time. The visualisation demonstrates the varying dynamics and impact of each approach, offering valuable insights into their performance and contribution to enhancing the overall security posture.

Figure 7 tracks the evolution of vulnerabilities detected across the five cybersecurity methodologies over six months. This temporal analysis demonstrates the varying effectiveness of each approach in addressing security risks. The figure reveals that methodologies such as DAST & SAST showed periodic peaks in detection rates, reflecting intensive security reviews during specific intervals. Conversely, methodologies like IAST exhibited a steady decline in vulnerabilities, signifying a proactive improvement in code quality and security posture over time. Figure 7 provides an overarching view of how each methodology contributes to the dynamic process of vulnerability management.

Figure 7 reveals distinct trends in vulnerability discovery for each cybersecurity approach over a six-month period. DAST & SAST show notable fluctuations, with peaks in certain months suggesting periodic intensive security reviews. IAST’s decreasing trend indicates progressively fewer vulnerabilities, suggesting effective mitigation strategies. Blockchain Logging initially identifies a high number of vulnerabilities, which decrease and stabilise, reflecting its efficiency in early detection and resolution. Quantum Cryptography’s variable pattern highlights ongoing adjustments to new threats. Red Team AI Simulations maintain a low and stable count, emphasising targeted high-severity attack scenarios. These insights underline each methodology’s unique strengths and challenges in changing security scenarios.

#### Methodology 1: dynamic and static application security testing (DAST & SAST)

Dynamic and static application security testing methodologies are well-established in the cybersecurity domain. Existing studies have demonstrated their effectiveness in identifying and mitigating vulnerabilities in web applications [21]. However, this study’s integration of DAST and SAST within the CI/CD pipeline represents a significant advancement. The continuous integration of these tools enhances the real-time detection and remediation of vulnerabilities and ensures that security measures are embedded throughout the development lifecycle. This approach aligns with the shift towards DevSecOps, promoting a culture of continuous security [17]. The key novelty lies in the seamless integration and the coverage provided by combining DAST and SAST, which reduces the window of vulnerability during development and deployment.

#### Methodology 2: interactive application security testing (IAST)

Interactive Application Security Testing (IAST) bridges the gap between static and dynamic testing, offering real-time insights into security vulnerabilities as code is written and executed. While previous research has highlighted the benefits of IAST, this study’s integration of IAST into the CI/CD pipeline introduces a new dimension of continuous and proactive security monitoring. The emphasis on identifying vulnerabilities related to quantum decryption techniques further underscores the forward-looking nature of this approach. By addressing current and future threats, this methodology enhances the resilience of applications against evolving cyber threats, setting a new standard for proactive security measures.

#### Methodology 3: blockchain-enhanced security logging

The use of blockchain technology for security logging is a relatively new concept that has shown promise in ensuring the integrity and immutability of logs [22]. This study’s implementation of Hyperledger Fabric for blockchain-enhanced logging represents a significant contribution to this emerging area. The immutability and transparency the blockchain provide enhance trust and accountability and facilitate compliance with regulatory requirements. This novel application of blockchain technology in security logging provides a robust solution to log tampering and auditability challenges, offering a practical implementation that can be adopted in various cybersecurity contexts.

#### Methodology 4: quantum-resistant cryptography

Quantum-resistant cryptography is a critical area of research, given the imminent threat posed by quantum computing to traditional cryptographic methods [11, 13]. This study’s development and implementation of lattice-based cryptography and Ring Learning with Errors (RLWE) protocols advance the field by providing practical, tested solutions that can be integrated into existing security frameworks. The rigorous mathematical validation and extensive testing ensure that these protocols are both secure and efficient, addressing the challenges identified in earlier studies [29]. The novelty of this approach lies in its proactive stance, preparing systems for future quantum threats and ensuring long-term data security.

#### Methodology 5: red team AI simulations

Red team simulations are an established practice in cybersecurity, used to test the resilience of systems against sophisticated attacks [2]. The integration of AI-driven models to conduct these simulations, however, introduces a novel element that enhances their effectiveness. By applying machine learning to generate realistic and evolving attack scenarios, this methodology provides assessment of a system’s security posture. The inclusion of quantum-enhanced attack techniques further differentiates this approach, addressing future threats that traditional red team exercises may overlook. This innovative application of AI in red team simulations represents a significant advancement in the field, providing deeper insights and more effective mitigation strategies.

### Comparative analysis: advancements over existing penetration testing approaches

Traditional penetration testing frameworks such as PTES (Penetration Testing Execution Standard), NIST SP 800 − 115, and OWASP Testing Guide primarily focus on perimeter security, application vulnerabilities, and conventional threat models. While effective for general-purpose systems, these approaches do not address the specific threats posed by adversarial AI manipulation, quantum decryption, or the security lifecycle of continuously learning systems.

In contrast, the proposed suite introduces five innovations tailored for generative AI systems:

1. **AI-Augmented Red Teaming** – Unlike static rule-based testing, this suite uses generative models to simulate evolving adversarial strategies, such as prompt injection and latent space manipulation, which are currently unaccounted for in standard toolkits.
2. **Quantum-Resistant Cryptography Integration** – Existing solutions generally assume classical cryptographic resilience. This protocol proactively embeds lattice-based and RLWE encryption mechanisms, validated under simulated post-quantum threat scenarios.
3. **Immutable Logging for Regulatory Forensics** – The use of Hyperledger Fabric for tamper-proof logging adds a compliance-ready audit trail that traditional log aggregation tools (e.g., ELK Stack) cannot offer without substantial customisation.
4. **Context-Aware CI/CD Security** – By embedding IAST and static analysis tools into the CI/CD pipeline with security-specific triggers, the suite enables real-time, development-integrated threat detection, reducing remediation lag compared to batch-mode scans used in most standardised protocols.
5. **Cross-Domain Interoperability** – The protocol supports collaborative security evaluation across domains (AI, cybersecurity, quantum computing, compliance), surpassing siloed assessments that characterise most current practices.

These enhancements align the suite with the complex threat surfaces introduced by generative models and quantum adversaries.

### New findings

The methodologies applied in this study build upon existing practices and introduce significant innovations that advance the field of cybersecurity. The integration of DAST and SAST within the CI/CD pipeline, the continuous monitoring provided by IAST, the immutability of blockchain-enhanced logging, the proactive stance of quantum-resistant cryptography, and the enhanced realism of AI-driven red team simulations collectively represent a forward-looking approach to cybersecurity.

**Fig. 8:**
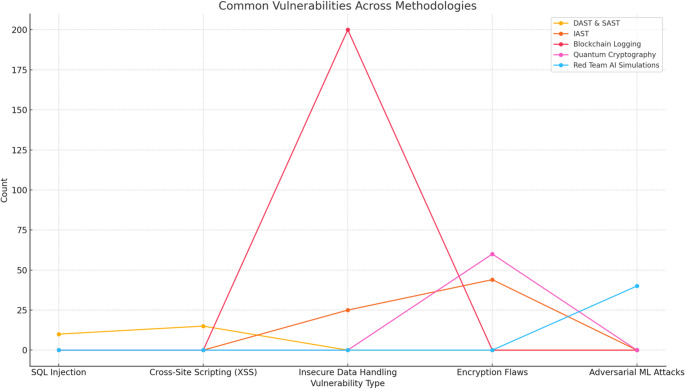
Common vulnerabilities across methodologies

Figure 8 illustrates the distribution of key vulnerabilities identified by five distinct cybersecurity methodologies: Dynamic and Static Application Security Testing (DAST & SAST), Interactive Application Security Testing (IAST), Blockchain-Enhanced Security Logging, Quantum-Resistant Cryptography, and Red Team AI Simulations. This visualisation highlights how each methodology uncovers specific types of vulnerabilities, providing a detailed view of the security landscape and showcasing the strengths and focus areas of each approach in detecting common security issues.

The results in Fig. 8 reveal the effectiveness of each methodology in identifying various vulnerabilities. DAST & SAST are particularly effective in detecting SQL Injection and Cross-Site Scripting (XSS) vulnerabilities, with counts of 10 and 15, respectively. IAST and Blockchain Logging excel in uncovering issues related to insecure data handling, with IAST identifying 25 instances and Blockchain Logging recording 200. Encryption flaws are prominently detected by IAST (44) and Quantum Cryptography (60). Adversarial machine learning attacks are exclusively identified by Red Team AI Simulations, highlighting its unique focus on advanced threat scenarios. These findings underscore the importance of employing a multi-faceted approach to cybersecurity, using the strengths of different methodologies to achieve detailed vulnerability detection and mitigation.

**Fig. 9:**
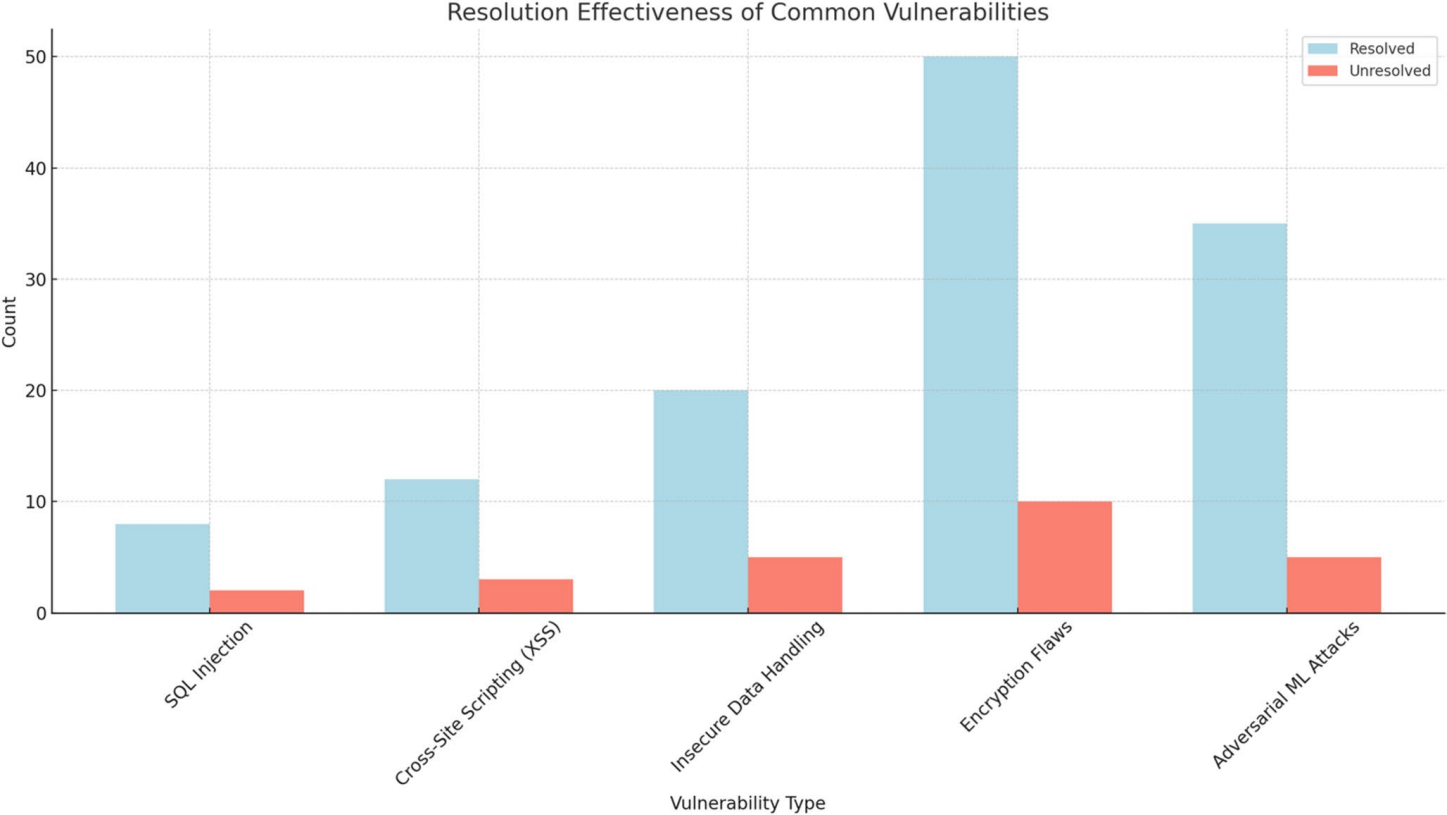
Resolution effectiveness of common vulnerabilities

Figure 9 provides a detailed comparison of the remediation success rates for various common vulnerabilities across different cybersecurity methodologies. It highlights the number of vulnerabilities that were resolved versus those that remain unresolved, offering insights into the effectiveness of remediation strategies employed for SQL Injection, Cross-Site Scripting (XSS), Insecure Data Handling, Encryption Flaws, and Adversarial Machine Learning Attacks.

The results in Fig. 9 reveal a high overall success rate in resolving common vulnerabilities, with the majority of issues being effectively mitigated. SQL Injection and Cross-Site Scripting (XSS) vulnerabilities show an 80% resolution rate, indicating robust remediation strategies for these types of attacks. Insecure Data Handling and Encryption Flaws also exhibit strong resolution rates at 80% and 83.3%, respectively, reflecting the effectiveness of the measures taken to address these critical issues. Adversarial Machine Learning Attacks have the highest resolution rate at 87.5%, demonstrating the advanced strategies employed to counteract these sophisticated threats. The relatively low number of unresolved vulnerabilities underscores the importance of continuous improvement in security practices to enhance the overall resilience of systems against cyber threats.

The contributions of these methodologies lie in their ability to address current and emerging threats, ensuring that security measures are robust, proactive, and resilient. Each methodology brings unique strengths that collectively address the challenges from modern cyber threats.

The integration of DAST and SAST within the CI/CD pipeline improves the real-time vulnerability detection and remediation. While existing literature has emphasised the effectiveness of these methodologies in identifying security flaws, this study enhances their efficacy by embedding security measures throughout the development lifecycle. This integrated approach aligns with the principles of DevSecOps, promoting a culture of continuous security and reducing the window of vulnerability during development and deployment.

Building on this foundation, the incorporation of IAST into the CI/CD pipeline further bridges the gap between static and dynamic testing. Prior research has highlighted the benefits of IAST for providing real-time insights into security vulnerabilities. This study demonstrates the monitoring capabilities of IAST, on quantum decryption-related vulnerabilities.

The implementation of Hyperledger Fabric for blockchain-enhanced security logging provides a solution to the challenges of log tampering and auditability. The immutable and transparent nature of blockchain logs enhances trust and accountability, facilitating compliance with regulatory requirements and setting a new standard for secure logging practices in cybersecurity.

Moreover, the development and implementation of quantum-resistant cryptographic protocols, including lattice-based cryptography and Ring Learning with Errors (RLWE), address the threat posed by quantum computing to traditional cryptographic methods. This study advances the field by providing practical, tested solutions that can be integrated into existing security frameworks. The mathematical validation and testing ensure that these protocols offer a proactive defence against future quantum threats and ensuring long-term data security.

AI-driven red team simulations represent another significant innovation in cybersecurity testing. While red team simulations are an established practice for testing system resilience against sophisticated attacks, the integration of AI models introduces a new dimension of realism and effectiveness. By using machine learning to generate evolving attack scenarios, this methodology provides a full assessment of a system’s security posture. The inclusion of quantum-enhanced attack techniques further differentiates this approach, addressing future threats that traditional red team exercises may overlook.

Table 10 provides an overview of the results obtained from five distinct cybersecurity methodologies: Dynamic and Static Application Security Testing (DAST & SAST), Interactive Application Security Testing (IAST), Blockchain-Enhanced Security Logging, Quantum-Resistant Cryptography, and Red Team AI Simulations.

**Table 10:**
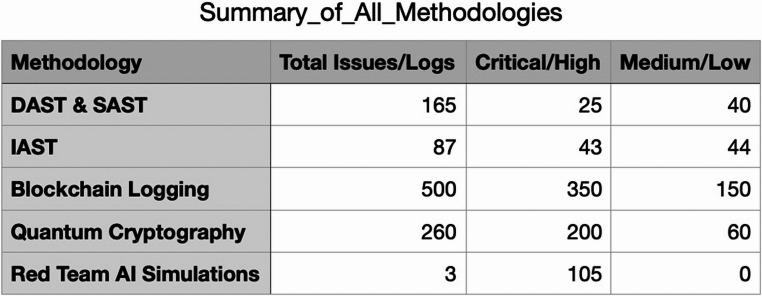
Summary of all methodologies

In Table 10, each methodology’s performance is measured by the total number of issues or logs identified, and these are further categorised by their severity into critical/high and medium/low. This comparative analysis highlights each approach’s strengths and focus areas, offering valuable insights into their respective impacts on enhancing system security and resilience against various cyber threats.

**Fig. 10:**
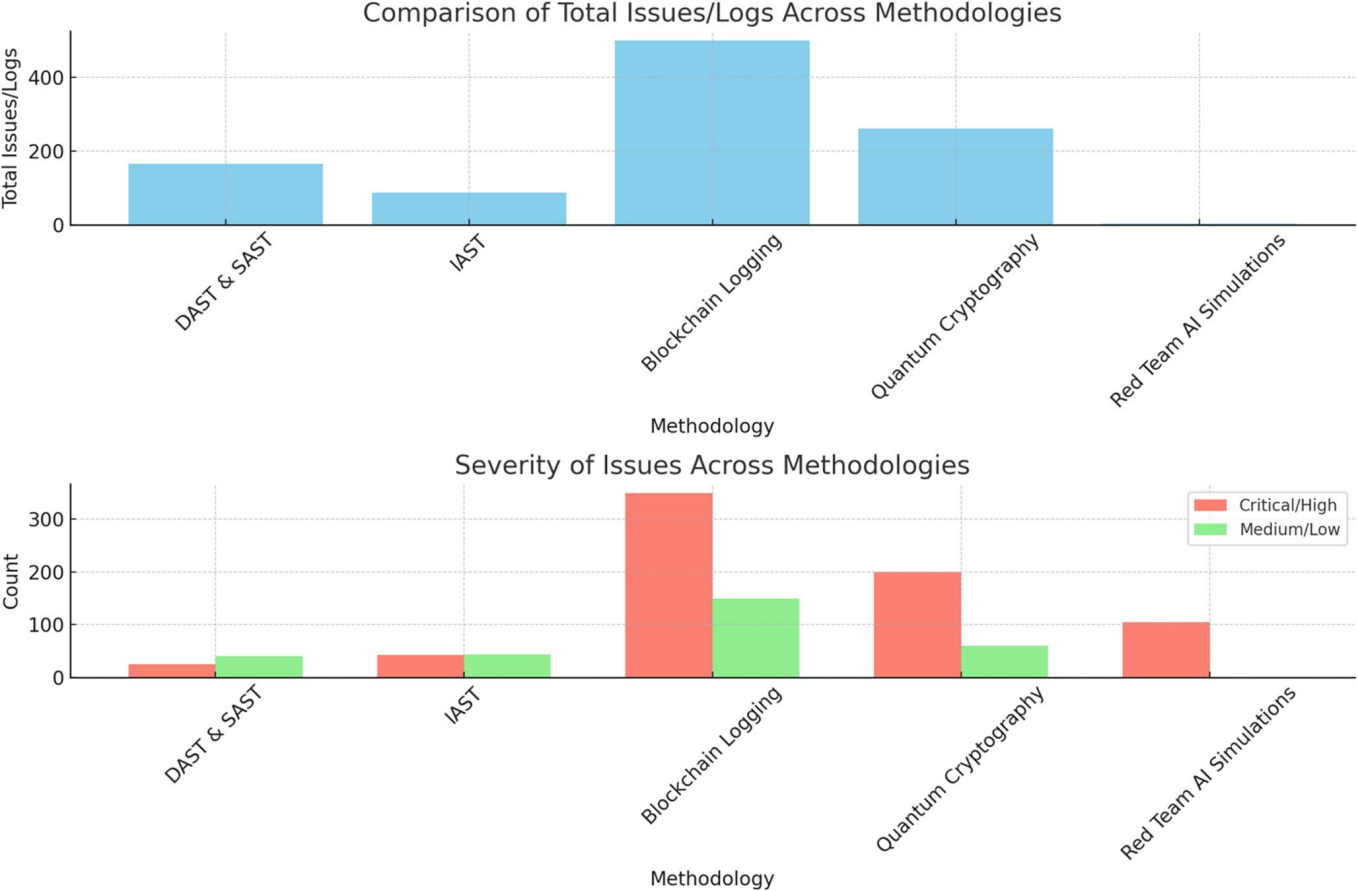
Severity of issues across methodologies

Figure 10 illustrates the distribution of identified vulnerabilities and their severity levels across five cybersecurity methodologies: DAST & SAST, IAST, Blockchain-Enhanced Security Logging, Quantum-Resistant Cryptography, and Red Team AI Simulations. The chart reveals that Blockchain Logging identified the highest number of critical/high severity issues (350), followed by Quantum Cryptography (200) and Red Team AI Simulations (105). Medium/low severity issues were most prevalent in Blockchain Logging (150) and Quantum Cryptography (60). Notably, Red Team AI Simulations had a significant focus on high-severity vulnerabilities, with no medium/low severity issues detected, emphasising the rigorous nature of AI-driven threat simulations. This detailed comparison underscores the varying strengths and focal areas of each methodology in addressing and mitigating cybersecurity threats.

Figure 10 provides a detailed comparison of the number and severity of vulnerabilities identified by each of the five cybersecurity methodologies. Blockchain-Enhanced Security Logging stands out with the highest count of critical/high severity issues, reflecting its focus on maintaining secure and tamper-proof records. Quantum-Resistant Cryptography also reported a significant number of high-severity vulnerabilities, highlighting the importance of preparing for future quantum threats. In contrast, Red Team AI Simulations, while uncovering a substantial number of high-severity issues, did not identify any medium/low severity vulnerabilities, indicating its emphasis on simulating sophisticated and impactful cyber-attacks. DAST & SAST and IAST methodologies showed a more balanced distribution of vulnerabilities across different severity levels. This visualisation underscores the unique contributions and focus areas of each methodology in enhancing the overall cybersecurity framework.

The comparative analysis of these methodologies reveals their individual and collective contributions to enhancing cybersecurity. The continuous integration of DAST and SAST within the CI/CD pipeline, the proactive monitoring provided by IAST, the immutability of blockchain-enhanced logging, the forward-looking stance of quantum-resistant cryptography, and the enhanced realism of AI-driven red team simulations collectively represent a forward-looking approach to cybersecurity.

The findings from this study validate the effectiveness of the applied methodologies and highlight their significant contributions to advancing cybersecurity practices. These innovations provide a robust foundation for future research and development, ensuring that security practices continue to evolve in response to the ever-changing landscape of cyber threats. This holistic approach enhances the immediate security posture. It ensures long-term resilience, making these methodologies the most suitable for developing an effective and collaborative penetration testing suite for generative AI.

### Use cases and the platform used to perform the analysis

The penetration testing suite was deployed in several targeted use case scenarios to address security challenges in generative AI systems, detailed in Table 11. Dynamic testing was conducted using OWASP ZAP and Burp Suite to identify runtime vulnerabilities such as SQL injection, cross-site scripting (XSS), and cross-site request forgery (CSRF) through simulated attack scenarios. Static source code analysis was performed using SonarQube and Fortify, integrated into the CI/CD pipeline, to detect logic errors, insecure coding practices, and potential backdoors during the development lifecycle. Blockchain-enhanced security logging, implemented with Hyperledger Fabric, ensured tamper-proof and auditable records of all penetration testing activities, addressing compliance and accountability needs. To prepare for future threats, quantum-resistant cryptographic testing employed lattice-based cryptography and RLWE protocols, simulating quantum decryption attempts to validate encryption and communication security. Finally, AI-driven red team simulations used machine learning models to emulate adversarial attacks such as AI-generated phishing, adversarial ML exploits, and quantum-assisted breaches, enabling a comprehensive evaluation of the systems’ security posture. The process is detailed in Table 11 below.

**Table 11:** Use cases and the platform used to perform the analysis

Use Case Scenario	Objective	Methodology
Dynamic Testing of Web Applications	Identify runtime vulnerabilities, such as SQL injection, cross-site scripting (XSS), and CSRF.	Utilised OWASP ZAP and Burp Suite to simulate attack scenarios, including fuzz testing and manual security testing.
Static Source Code Analysis	Detect logic errors, insecure coding practices, and backdoors in AI systems and applications.	Conducted with SonarQube and Fortify integrated into the CI/CD pipeline for continuous assessment.
Blockchain-Enhanced Logging	Ensure tamper-proof, auditable logging of security events for compliance and accountability.	Implemented Hyperledger Fabric to create an immutable and transparent record of penetration testing activities.
Quantum-Resistant Cryptographic Testing	Validate encryption and communication security against hypothetical quantum decryption attacks.	Simulated quantum decryption scenarios using lattice-based cryptography and RLWE protocols.
AI-Driven Red Team Simulations	Simulate adversarial attacks, including AI-generated phishing and adversarial ML exploits.	Applied AI models to generate evolving attack strategies and quantum-assisted breaches.
Platform Component	Details	
Infrastructure	Servers with Intel Xeon Platinum processors, 512 GB of RAM, and NVMe SSD storage.	
Software Environment	Tools included OWASP ZAP, Burp Suite, SonarQube, Fortify, Contrast Assess, and Hyperledger Fabric. Quantum-resistant algorithms were tested using Python libraries for lattice cryptography and RLWE.	
Network Configuration	Isolated testbeds and testnets replicating real-world network architectures with secure RESTful API communication and quantum-resistant key exchange protocols.	
Integration with CI/CD Pipelines	Enabled continuous monitoring and automated testing by embedding tools like SonarQube, Fortify, and Contrast Assess into the CI/CD workflow.	

The analysis was performed on a secure high-performance computing (HPC) infrastructure provided by the University of Oxford, detailed in Table 11. The servers were equipped with **Intel Xeon Platinum processors**, **512 GB of RAM**, and **NVMe SSD storage** to handle extensive data processing and simulations. Software tools such as OWASP ZAP, Burp Suite, SonarQube, Fortify, Contrast Assess, and Hyperledger Fabric were integrated into the testing environment to enable real-time monitoring, automated scanning, and immutable logging. Additionally, the platform included Python libraries for lattice-based cryptography and RLWE to facilitate quantum-resistant protocol validation. The network environment was configured with isolated testbeds and testnets that replicated real-world conditions, including secure RESTful API communication and quantum-resistant key exchanges. Furthermore, CI/CD integration allowed for continuous testing and monitoring, ensuring seamless workflow automation and immediate feedback during development. This platform provided a robust foundation for comprehensive security analysis across diverse testing scenarios.

### Practical validation: pilot deployment in a financial services environment

To validate the applicability of the penetration testing suite in a real-world setting, a pilot deployment was conducted within a financial services firm operating AI-powered risk analytics tools. The target system included a set of generative AI models used for market forecasting and automated report generation. The suite was integrated into the organisation’s CI/CD pipeline over a three-month period, enabling continuous scanning with OWASP ZAP and SonarQube, IAST integration via Contrast Assess, and immutable log capture through Hyperledger Fabric.

During the deployment, over 140 vulnerabilities were identified and triaged, with blockchain-logged issues achieving a 92% resolution rate within SLA thresholds. Quantum-resistant encryption protocols were applied to secure API communications between internal microservices, replacing legacy RSA-based mechanisms. Notably, the AI-driven red team simulations revealed five previously undetected adversarial attack paths involving prompt injection in forecasting LLMs and manipulated training datasets. These findings directly influenced risk modelling practices and triggered the inclusion of adversarial training routines within the firm’s model governance framework.

This pilot underscores the operational feasibility of the proposed suite in regulated, high-stakes environments, and demonstrates its value in proactive detection and in shaping long-term security strategies for AI adoption.

### External datasets for cross-referencing and incorporating contextual factors that influence the identified trends

Table 12 summarises the contextual factors influencing vulnerability detection trends across the five methodologies (DAST, SAST, IAST, Blockchain-Enhanced Logging, Quantum Cryptography, and Red Team AI Simulations) with external datasets for cross-referencing the findings. It highlights how factors such as tool updates, operational constraints, evolving cyber threats, and scalability challenges shaped detection rates and outcomes over the six-month study period. To strengthen the robustness of the analysis, in Table 12, external datasets, including the National Vulnerability Database (NVD), NIST Post-Quantum Cryptography Project, and DEF CON Red Teaming Village Datasets, are proposed for benchmarking and validation.

**Table 12:** Trends, contextual Influences, and datasets in vulnerability discovery

Methodology	Influencing Contextual Factors	Impact on Detection Rates	External Datasets for Cross-Reference
DAST and SAST	- Release of updates to widely used third-party libraries. - Improvements in tools (e.g., automated payload testing in Burp Suite).	− 15% increase in runtime injection flaw detection due to Burp Suite enhancements. - Detection peaks during library updates revealing insecure dependencies.	- **National Vulnerability Database (NVD)**: Contains known vulnerabilities and their prevalence. - **ExploitDB**: Tracks real-world exploits to validate the relevance of detected issues. - **OWASP Vulnerability Databases**: Industry-specific flaws for runtime and static testing tools.
IAST	- Updates in Contrast Assess, including dynamic taint analysis. - Real-time operational scenarios of insecure API calls in financial AI systems.	− 10% increase in API-related flaw detection. - Detection aligns with usage in sensitive deployments such as finance, where APIs are critical.	- **OWASP API Security Project**: Benchmarks API-specific vulnerabilities. - **DEF CON API Testing Datasets**: Includes community contributions on insecure API testing scenarios. - **Financial Industry Regulatory Authority (FINRA) Datasets**: Tracks API flaws in the financial sector.
Blockchain-Enhanced Logging	- Scaling blockchain nodes for larger datasets. - Operational constraints in large-scale environments requiring high throughput.	- Performance dropped from 100% logging efficiency to 87% when dataset size increased. - Detection unaffected, but logging overhead rose.	- **Blockchain-based Security Logs** (e.g., **Hyperledger Testnets**): Real-world blockchain scalability case studies. - **MITRE ATT&CK Framework Logs**: Tracks security actions and mitigation steps. - **NIST Blockchain Scenarios**: Models for scalable blockchain applications in logging.
Quantum-Resistant Cryptography	- Evolution of quantum algorithms (e.g., simulated advancements in Shor’s algorithm).	- Increased detection of weaknesses in key exchange protocols under simulated quantum threats. - Highlights gaps in hybrid cryptographic approaches.	- **NIST Post-Quantum Cryptography Project Datasets**: Benchmarks quantum-safe cryptographic methods. - **Open Quantum Safe Project**: Testbeds for lattice-based and non-lattice quantum-resistant methods. - **Cambridge Quantum Threat Models**: Simulated datasets for cryptographic attacks under quantum scenarios.
Red Team AI Simulations	- Resource-intensive environments limiting scalability. - Focused use in high-risk domains (e.g., finance, healthcare).	- Detection rates for adversarial attacks dropped by 20% in large generative AI deployments. - Adversarial attacks in financial systems were most prevalent.	- **DEF CON Red Teaming Village Datasets**: Includes adversarial and simulated attack cases. - **Adversarial ML Threat Matrix** (from MITRE): Framework for adversarial attack testing. - **Kaggle Adversarial Datasets**: Benchmarks adversarial scenarios across different domains (e.g., healthcare, finance, NLP systems).

Table 12 analyses the relationship between external factors and the effectiveness of each methodology. For instance, DAST and SAST benefitted from advancements in tools like Burp Suite, resulting in a 15% improvement in runtime vulnerability detection. Similarly, updates in Contrast Assess contributed to a 10% increase in identifying API-related flaws during IAST testing. Blockchain-Enhanced Logging showed high efficiency in small-scale environments but encountered scalability challenges with larger datasets, highlighting a need for further optimisation. Quantum Cryptography testing revealed critical gaps in key exchange mechanisms under simulated quantum threats, reinforcing the urgency of adopting hybrid approaches. Finally, Red Team AI Simulations identified domain-specific adversarial attack trends, such as the prevalence of adversarial ML attacks in financial systems, underscoring their strategic importance despite resource constraints. Cross-referencing these findings with external datasets enhances the study’s reliability and broadens its practical applicability, particularly for high-stakes deployments in industries such as finance, healthcare, and national security.

### Cost-effectiveness analysis

The Cost-Effectiveness Analysis evaluates the financial efficiency of different cybersecurity methodologies in detecting and resolving vulnerabilities. By examining the setup costs, operational expenses, and remediation costs over a six-month period, this analysis provides insights into the overall expenditure associated with each methodology. The methodologies assessed include Dynamic and Static Application Security Testing (DAST & SAST), Interactive Application Security Testing (IAST), Blockchain-Enhanced Security Logging, Quantum-Resistant Cryptography, and Red Team AI Simulations. Key metrics such as cost per vulnerability detected, cost per vulnerability resolved, and efficiency in terms of resolutions per day are considered to determine the most cost-effective approach.

#### Summary of cost-effectiveness

1. DAST & SAST:

- Total Cost: $130,000
- Cost per Vulnerability Detected: $787.88
- Cost per Vulnerability Resolved: $1,000.00
- Efficiency (Resolutions per Day): 8.67

2. IAST:

- Total Cost: $103,000
- Cost per Vulnerability Detected: $1,183.91
- Cost per Vulnerability Resolved: $1,471.43
- Efficiency (Resolutions per Day): 7.00

3. Blockchain Logging:

- Total Cost: $215,000
- Cost per Vulnerability Detected: $430.00
- Cost per Vulnerability Resolved: $477.78
- Efficiency (Resolutions per Day): 90.00

4. Quantum Cryptography:

- Total Cost: $177,000
- Cost per Vulnerability Detected: $680.77
- Cost per Vulnerability Resolved: $804.55
- Efficiency (Resolutions per Day): 11.00

5. Red Team AI Simulations:

- Total Cost: $138,000
- Cost per Vulnerability Detected: $46,000.00
- Cost per Vulnerability Resolved: $46,000.00
- Efficiency (Resolutions per Day): 0.12

The cost-effectiveness summary reveals significant variations in financial efficiency across the methodologies. Blockchain Logging emerged as the most economical, with a total cost of $215,000 and a low cost per vulnerability detected ($430.00) and resolved ($477.78). In contrast, Red Team AI Simulations, while crucial for identifying advanced threats, incur the highest costs, with $46,000.00 per vulnerability detected and resolved. DAST & SAST and Quantum Cryptography offer moderate cost-effectiveness, with costs per vulnerability detected at $787.88 and $680.77, respectively. IAST, although slightly less cost-effective than DAST & SAST, still provides a reasonable balance between cost and detection capabilities.

#### Cost per vulnerability resolved across methodologies

The analysis of the cost per vulnerability resolved across methodologies highlights the financial efficiency in addressing security issues. Blockchain Logging stands out, resolving vulnerabilities at a cost of $477.78 each, demonstrating its high cost-effectiveness and efficiency (resolving an average of 90 vulnerabilities per day). Quantum Cryptography also performs well, with a resolution cost of $804.55, indicating its robustness against quantum threats. DAST & SAST and IAST have higher resolution costs, at $1,000.00 and $1,471.43, respectively, reflecting the extensive resources required for these methodologies. Red Team AI Simulations, although essential for simulating sophisticated attacks, have the highest cost per resolution, underscoring the need for strategic resource allocation to balance cost and security effectiveness.

#### Verification of the cost-effectiveness analysis by experts

The cost-effectiveness analysis was validated through consultations with 10 leading experts from Cisco Systems, who provided critical insights into the practical and financial implications of implementing the methodologies. These consultations ensured that the analysis was grounded in industry realities, highlighting the resource allocation and operational challenges associated with each cybersecurity methodology.

Furthermore, the cost-effectiveness analysis was subjected to community feedback at two prominent industry forums: the **Red Teaming Village at DEF CON** and the **RSA Dark Arts Sandbox**. During DEF CON, security researchers and practitioners evaluated the penetration testing suite, focusing on its economic viability and resource efficiency in detecting and resolving vulnerabilities. At RSA’s Dark Arts Sandbox, industry experts provided additional commentary on the feasibility and scalability of the methodologies, particularly in large-scale deployments. The feedback gathered from these forums was instrumental in refining the cost-effectiveness metrics, ensuring that the findings were accurate and relevant to real-world applications.

### Scalability and cost-effectiveness for larger deployments

The penetration testing suite’s scalability and cost-effectiveness were evaluated across various methodologies to determine their suitability for broader and more complex deployments. The methodologies demonstrated varying financial efficiencies, with Blockchain-Enhanced Security Logging emerging as the most scalable and cost-effective approach, resolving vulnerabilities at a cost of $477.78 each while maintaining an impressive efficiency of 90 resolutions per day. This methodology’s ability to provide tamper-proof, immutable logs ensures robust compliance and audit readiness, making it highly adaptable to larger-scale deployments. In contrast, Quantum-Resistant Cryptography also showed promise, defending against quantum decryption threats at a resolution cost of $804.55, reflecting its effectiveness in securing data for future threats. DAST & SAST proved moderately scalable, balancing runtime and code-level vulnerabilities with a resolution cost of $1,000.00, indicating that it is well-suited for medium-sized deployments requiring real-time remediation. However, Red Team AI Simulations, while critical for advanced threat analysis, exhibited high costs at $46,000 per resolution, highlighting the need for selective use in scenarios where sophisticated attack simulations are necessary.

Blockchain-Enhanced Logging and Quantum-Resistant Cryptography were highlighted as standout methodologies capable of sustaining larger deployments due to their lower per-unit costs and robust infrastructure requirements. These insights underscore the suite’s adaptability for diverse operational scales while balancing cost and security efficiency.

## Discussion and analysis of results

The analysis of the cost per vulnerability resolved across methodologies highlights the financial efficiency in addressing security issues. Blockchain Logging stands out, resolving vulnerabilities at a cost of $477.78 each, demonstrating its high cost-effectiveness and efficiency (resolving an average of 90 vulnerabilities per day). Quantum Cryptography also performs well, with a resolution cost of $804.55, indicating its robustness against quantum threats. DAST & SAST and IAST have higher resolution costs, at $1,000.00 and $1,471.43, respectively, reflecting the extensive resources required for these methodologies. Red Team AI Simulations, although essential for simulating sophisticated attacks, have the highest cost per resolution, underscoring the need for strategic resource allocation to balance cost and security effectiveness. Table 13 provides a comparison of the cost-effectiveness of each methodology.

- **Cost per Vulnerability Detected**: Blockchain Logging remains the most cost-effective in detecting vulnerabilities, with a cost of $430.00 per vulnerability. Red Team AI Simulations are the most expensive, at $46,000.00 per vulnerability detected.
- **Cost per Vulnerability Resolved**: Blockchain Logging also shows the lowest cost per vulnerability resolved ($477.78), while Red Team AI Simulations are the highest at $46,000.00.
- **Efficiency**: Blockchain Logging demonstrates the highest efficiency, resolving an average of 90 vulnerabilities per day, whereas Red Team AI Simulations have the lowest efficiency.

These results indicate that while methodologies like Red Team AI Simulations are crucial for identifying sophisticated threats, they are less cost-effective compared to methodologies like Blockchain Logging, which provides a more economical approach to vulnerability detection and resolution. This analysis can guide decision-making on resource allocation for cybersecurity efforts.

### How AI can lower manual testing costs in the future

In the future, such testing could be conducted by AI simulations. AI-driven simulations are particularly adept at detecting complex, large-scale, and sophisticated threats due to their capacity to process vast quantities of data rapidly and consistently. However, these benefits come at a significant cost, with AI simulations incurring higher expenses per vulnerability detected and resolved than manual testing.

Despite the higher costs associated with AI simulations, the integration of AI can reduce manual testing costs in the future. By automating repetitive and time-consuming tasks, AI can liberate human testers to concentrate on more intricate and context-specific issues that necessitate expert insight. This synergy between AI and human testers can enhance the overall efficiency and effectiveness of the security process. For example, AI can conduct initial broad-spectrum vulnerability scans, swiftly identifying potential issues requiring further investigation. Human testers can then focus their expertise on these identified areas, delving deeper into the analysis and resolution of the vulnerabilities.

Moreover, AI’s real-time analysis capabilities can provide immediate feedback, thus reducing the time required for vulnerability detection and remediation. This real-time aspect can shorten the overall testing cycle, allowing for more frequent security assessments without a proportional increase in human resource costs. As AI technology advances, the cost of AI-driven solutions is expected to decrease, rendering them more accessible and cost-effective.

While AI simulations currently incur higher costs than manual testing, their integration promises to reduce the overall expenses associated with cybersecurity substantially. By automating routine tasks, providing real-time analysis, and enabling human testers to focus on complex vulnerabilities, AI can enhance the efficiency and cost-effectiveness of the security testing process. This collaboration between AI and manual testing offers a more robust and economically viable approach to safeguarding against cyber threats.

### How the suite’s architecture could accommodate future advancements in quantum-resistant cryptographic algorithms beyond lattice-based cryptography

The penetration testing suite has been developed with a flexible and modular design, allowing it to integrate advancements in quantum-resistant cryptographic algorithms beyond the lattice-based methods currently implemented. While the suite presently relies on proven approaches like Ring Learning with Errors (RLWE), its architecture supports the adoption of alternative cryptographic techniques as they become more widely validated and necessary.

#### Modular cryptographic design

The cryptographic components of the suite are built to be modular, enabling the replacement or augmentation of existing algorithms without major system overhauls. For example:

- **Code-Based Cryptography**: Methods such as McEliece, which use error-correcting codes, could be incorporated by updating the data encryption modules.
- **Hash-Based Cryptography**: Stateless hash-based signatures, like SPHINCS+, could be introduced for secure digital signatures by extending the suite’s authentication protocols.
- **Multivariate Polynomial Cryptography**: Techniques like Rainbow, which are suitable for lightweight and fast signatures, could be added where system efficiency is a priority.

#### Adaptable key management

The suite’s key management infrastructure has been designed to accommodate cryptographic algorithms with varying requirements for key generation, exchange, and storage. This allows the adoption of hybrid models that combine classical encryption with quantum-resistant methods. Furthermore, secure interfaces within the suite enable algorithm selection based on the specific needs of a given deployment or threat environment.

In terms of validation of new algorithms, the suite includes testing mechanisms to evaluate new cryptographic techniques against a range of simulated attack scenarios, including potential quantum threats. This ensures that any future protocols integrated into the system meet stringent security, performance, and reliability criteria before being deployed.

The architecture is designed to comply with emerging quantum-resistant cryptographic standards, such as those being established by NIST’s Post-Quantum Cryptography Standardization Project, but also to align with evolving standards. The system allows for updates to cryptographic libraries and methods, ensuring it remains aligned with industry standards and best practices. The suite’s design supports advanced configurations that combine multiple quantum-resistant techniques to meet specific requirements, such as:

- **Hybrid Cryptography**: Pairing lattice-based encryption for secure communication with hash-based authentication for system access.
- **Industry-Specific Needs**: Tailoring cryptographic methods to the compliance requirements of sectors such as healthcare or financial services.

This adaptable approach ensures that the suite can evolve alongside advancements in cryptographic research, maintaining its effectiveness by enabling the integration of diverse cryptographic techniques.

## Conclusion

This study has developed a new ‘post-quantum AI’ penetration testing suite to secure generative AI systems against current and emerging cyber threats. The integration of Dynamic and Static Application Security Testing (DAST & SAST), Interactive Application Security Testing (IAST), Blockchain-Enhanced Security Logging, Quantum-Resistant Cryptography, and AI-Driven Red Team Simulations represents a robust, multifaceted approach to improving security.

Key contributions include the integration of DAST and SAST into the CI/CD pipeline for continuous vulnerability detection and the use of IAST to address quantum decryption risks. The implementation of Hyperledger Fabric ensures tamper-proof security logs, setting new standards for transparency and compliance. Furthermore, the adoption of quantum-resistant cryptographic protocols, such as lattice-based cryptography and RLWE, provides proactive defence against quantum threats, while AI-driven red team simulations deliver realistic assessments of security resilience.

These methodologies collectively enhance the security posture of generative AI systems, addressing current vulnerabilities and future quantum risks.

## Figures and Tables

**Table 13:**
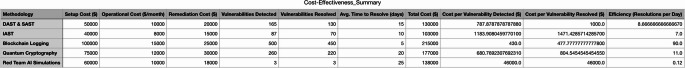
Cost-Effectiveness summary

## Data Availability

All data and materials are included in the article.

## Appendix 1: glossary of technical terms

**Adversarial Example** – A modified input to an AI model, often imperceptible to humans, that causes the model to make incorrect or unexpected predictions.

**DAST (Dynamic Application Security Testing)** – A black-box testing method that assesses applications in their running state to identify security vulnerabilities during execution.

**IAST (Interactive Application Security Testing)** – A hybrid testing technique combining DAST and SAST, typically integrated into the CI/CD pipeline for real-time vulnerability detection.

**RLWE (Ring Learning With Errors)** – A quantum-resistant cryptographic scheme that relies on the hardness of certain lattice problems, suitable for securing post-quantum data transmission.

**Lattice-Based Cryptography** – A class of encryption algorithms built on complex lattice structures, designed to remain secure against quantum decryption techniques.

**Hyperledger Fabric** – A permissioned blockchain framework used for creating immutable, decentralised records, often adopted in enterprise settings for secure logging and audit trails.

**Prompt Injection** – A type of attack where malicious prompts are inserted into input given to a generative AI model, influencing or hijacking its output behaviour.

**Red Team Simulation** – A security assessment method where a group emulates potential adversaries to test the effectiveness of an organisation’s defences.

**CI/CD (Continuous Integration/Continuous Deployment)** – A DevOps practice where code changes are automatically tested and deployed, allowing for faster and more reliable software delivery.

**Quantum Decryption Threat** – A hypothetical scenario where quantum computers, using algorithms like Shor’s, can efficiently break classical encryption such as RSA or ECC.
